# PRRSV hijacks DDX3X protein and induces ferroptosis to facilitate viral replication

**DOI:** 10.1186/s13567-024-01358-y

**Published:** 2024-08-18

**Authors:** Qian Mao, Shengming Ma, Shuangyu Li, Yuhua Zhang, Shanshan Li, Wenhui Wang, Fang Wang, Zekun Guo, Chengbao Wang

**Affiliations:** 1 College of Veterinary Medicine, Northwest Agriculture and Forestry University, Yangling, China; 2 Henan Joint International Research Laboratory of Veterinary Biologics Research and Application, Anyang Institute of Technology, Anyang, 455000 China; 3 College of Life Science, Northwest Agriculture and Forestry University, Yangling, China

**Keywords:** PRRSV, DDX3X, transcriptome, metabolome, ferroptosis

## Abstract

Porcine reproductive and respiratory syndrome virus (PRRSV) is a severe disease with substantial economic consequences for the swine industry. The DEAD-box helicase 3 (*DDX3X*) is an RNA helicase that plays a crucial role in regulating RNA metabolism, immunological response, and even RNA virus infection. However, it is unclear whether it contributes to PRRSV infection. Recent studies have found that the expression of *DDX3X* considerably increases in Marc-145 cells when infected with live PRRSV strains Ch-1R and SD16; however, it was observed that inactivated viruses did not lead to any changes. By using the RK-33 inhibitor or *DDX3X*-specific siRNAs to reduce DDX3X expression, there was a significant decrease in the production of PRRSV progenies. In contrast, the overexpression of *DDX3X* in host cells substantially increased the proliferation of PRRSV. A combination of transcriptomics and metabolomics investigations revealed that in PRRSV-infected cells, *DDX3X* gene silencing severely affected biological processes such as ferroptosis, the FoxO signalling pathway, and glutathione metabolism. The subsequent transmission electron microscopy (TEM) imaging displayed the typical ferroptosis features in PRRSV-infected cells, such as mitochondrial shrinkage, reduction or disappearance of mitochondrial cristae, and cytoplasmic membrane rupture. Conversely, the mitochondrial morphology was unchanged in DDX3X-inhibited cells. Furthermore, silencing of the *DDX3X* gene changed the expression of ferroptosis-related genes and inhibited the virus proliferation, while the drug-induced ferroptosis inversely promoted PRRSV replication. In summary, these results present an updated perspective of how PRRSV infection uses DDX3X for self-replication, potentially leading to ferroptosis via various mechanisms that promote PRRSV replication.

**Supplementary Information:**

The online version contains supplementary material available at 10.1186/s13567-024-01358-y.

## Introduction

Porcine reproductive and respiratory syndrome (PRRS) is a highly contagious swine disease that poses a serious threat to the global swine industry. It is caused by the porcine reproductive and respiratory syndrome virus (PRRSV) and presents clinical symptoms that include abortion in pregnant sows and respiratory disorders in piglets [1, 2]. As a member of the *Arteriviridae* family (*Nidovirales*), PRRSV is an enveloped and single-stranded positive RNA virus with a genome size of 15.4 kb [3, 4]. The epidemic PRRSV strains are typically categorised into two types: European or American, also known as genotypes 1 and 2. This classification is based on the antigenicity, genome, and pathogenicity [5–7].

The first Chinese strain, CH-1A, was reported in 1996 and was attenuated for producing the widely used Ch-1R vaccine [8, 9]. The SD16 strain is the highly pathogenic PRRSV (HP-PRRSV) that typically exhibits higher replication efficiency in vivo and in vitro than the classical Ch-1A strain. PRRSV establishes a persistent infection that remains for a long term in hosts without being eliminated by the immune system or drug therapy [10]. The chronic infection may be due to immunological failure resulting from virus-induced abnormalities in the host immune system [11, 12]. Another important characteristic of PRRSV infection is the effect of antibody-dependent enhancement (ADE), potentially making the virus more infectious and prevalent in pig populations with a low level of specific PRRSV antibodies. Furthermore, the wide diversity of PRRSV strains and their unique characteristics make disease control more challenging [13–17]. Although there have been documented breakthroughs in PRRSV research, the fundamental processes involved in PRRSV pathogenesis still require clarification.

The DEAD-box helicase 3 (DDX3X) is an RNA-dependent protein with nucleic acid unwinding activity. It performs important roles in multiple cellular processes, including transcriptional regulation, RNA metabolism and transport, RNA splicing, and post-transcriptional regulation [18–20]. Previous research has shown that certain RNA viruses may employ the driving force of DDX3X-mediated ATP hydrolysis—or its ability to construct large ribonucleoprotein complexes—as a means to facilitate different stages of the virus replication cycle, which may either limit or promote viral replication [18, 21–23]. For instance, the K7 protein of vaccinia virus (VACV) interacts with the DDX3X protein to inhibit the interferon pathway and assists the VACV proliferation in the host [24]. In the case of the hepatitis C virus (HCV), DDX3X can bind the viral core protein and silence DDX3X expression in HuH-7-derived cells, significantly suppressing the accumulation of both the genome-length HCV RNA and its replicon RNA [25, 26]. It has also been found that DDX3X can inhibit the reverse transcription of the hepatitis B virus (HBV) by incorporating HBV polymerase into the nucleocapsid, suggesting a potential role of DDX3X in HBV’s immune evasion [27].

Furthermore, DDX3X has been linked with immunomodulation to exert the type-I IFN-mediated antiviral immune response [28, 29]. Additionally, the protein can be recruited to CK1ε, where it interacts with DDX3X to phosphorylate Dv1, which initiates the Wnt signalling pathway after allosteric activation [30, 31]. Although previous studies have suggested a possible association between DDX3X and PRRSV’s Nsp2 protein, the exact mechanisms underlying this relationship remain unclear.

This study first evaluated the association between in vitro PRRSV infection and the expression of host cellular DDX3X. Subsequently, the activity of the DDX3X protein was modulated by chemical drug treatment, gene silencing, or overexpression to study its potential function in PRRSV infection and replication. A combination analysis of transcriptomics and metabolomics was performed to screen and validate the probable molecular mechanisms mediated by DDX3X in regulating PRRSV infection and replication. These results are expected to serve as a crucial theoretical foundation for future drug design and disease control.

## Materials and methods

### Cells and viruses

The Marc-145 cell line (5301MON-KCB08001YJ), derived from the embryonic kidney of African green monkeys, was used for PRRSV proliferation. The cells were maintained in Dulbecco’s Modified Eagle Medium (DMEM) culture medium containing 10% foetal bovine serum (FBS, Gibco, USA). They were cultivated at 37 ℃ in a 5% CO_2_ incubator.

The College of Veterinary Medicine at Northwest A&F University (China) provided two strains of PRRSV: the vaccine Ch-1R and SD16. These were propagated in Marc-145 cells by titrating to 50% tissue culture infective dose (TCID_50_). The inactivated PRRSVs were obtained by exposing the two virus stocks to 250-nm shortwave ultraviolet radiation (UV) overnight on ice in a 60-nm tissue culture dish, which was subsequently harvested and stored at − 80 °C.

### Virus infection

The Marc-145 cells were infected with PRRSV strains Ch-1R or SD16 at a multiplicity of infection (MOI) of 0.1. At 1 h post-incubation in serum-free DMEM medium, the cells were washed twice with phosphate-buffered saline (PBS) to remove unattached virus particles. Subsequently, the medium was replaced with a fresh DMEM maintenance medium containing 2% FBS. This was maintained until the cytopathic effects appeared at 48 to 72 h post-infection (hpi) for sample collection, for the analysis of DDX3X expression, and detection of virus-induced ferroptosis by western blot and real-time quantitative PCR (RT-qPCR).

### Cell treatment with RK-33 and virus infection

To inhibit the expression of DDX3X, a specific small molecule inhibitor, RK-33 (MedChemEpress, USA) was used [32]. For the IC_50_ assay, the Marc-145 cells were cultured in a 96-well plate for 24 h and then incubated with 2, 4, 6, 8, 10 or 12 μM of RK-33 for 72 h. Dimethyl sulfoxide (DMSO) served as the negative control. Cell viability was determined using a cell counting kit-8 assay (CCK-8 Beyotime, China) for detecting the optimal concentration of RK-33 without lesion to cells. Marc-145 cells were treated with RK-33 and infected with PRRSV strains Ch-1R and SD16. At 48 hpi, the cells were collected to analyse the expression of both DDX3X and viral genes.

### Knockdown or overexpression of DDX3X and virus infection

This study developed and synthesised three pairs of siRNAs that specifically target the *DDX3X* gene (Table 1) to promote the knockdown or overexpression of DDX3X. Furthermore, a plasmid (pEGFP-N1-DDX3X) capable of overexpressing DDX3X was constructed. The siRNAs (20 pmol each) and pEGFP-N1-DDX3X plasmid (1000 ng/well) were transfected into Marc-145 cells with Lipofectamine 3000 and incubated for 12 h, according to the manufacturer’s instructions.

**Table 1:** **siRNA target sequences**

Gene	Sequence (5′–3′)	
	Sense (5′–3′)	Antisense (5′–3′)
siRNA1	CCCUGCCAAACAAGCUAAUAU	AUAUUAGCUUGUUUGGCAGGG
siRNA2	CGUAAUGAUUCAAGAGGAAAGUCUA	UAGACUUUCCUCUUGAAUCAUUACG
siRNA3	GCUGAUGGAUGUUGGAUATT	UAUCCAACAUCCGAUCAGCTT

The cells were infected with PRRSV for 48 h before sample collection to analyse the subsequent DDX3X expression and to detect virus-induced ferroptosis by western blot and RT-qPCR. To activate and inhibit ferroptosis, Erastin (3 μM) and Ferrostatin-1 (2 μM) were used. Erastin is a ferroptosis inducer, which leads to reactive oxygen species (ROS) production and mitochondrial damage, thereby inducing intracellular ferroptosis [33]. The ferroptosis inhibitor, Ferrostatin-1, scavenges the iron-producing radical products and lipid peroxides [34].

### Western blot

The cell samples were collected and lysed using cell lysis buffer (RIPA, Beyotime, China) containing phenylmethylsulfonyl fluoride (PMSF, Beyotime, China) and phosphatase inhibitors. This buffer was used to extract proteins, which were then separated on 12.5% sodium dodecyl sulphate–polyacrylamide gel (SDS-PAGE) and transferred to nitrocellulose filter membranes (Boster Biological Technology, Ltd., China). The membranes were initially blocked for 3 h at room temperature using PBS containing 5% skimmed milk. They were then triple washed with PBS before overnight incubations with the primary antibodies PRRSV-N (Gene Tex, 1:5000, China), DDX3 (SANTA, 1:200, USA) or GAPDH (Gene Tex, 1:50 000) at 4 °C. After washing with PBS three times, the membrane was incubated for 2 h with horseradish peroxidase-conjugated Goat anti-Rabbit IgG and Goat anti-Mouse IgG (Beyotime, China) at room temperature. Protein bands were eventually observed using an enhanced chemiluminescence (ECL, Beyotime, China) detection system.

### Total RNA isolation and real-time quantitative PCR

The TRIzol reagent was used per the manufacturer’s instructions to extract the total cellular RNA. The EvoM-MLV Reverse Transcription Kit was used to reverse transcribe the total RNAs and create cDNA molecules. Prior to this, the genomic DNAs were extracted using DNase digestion. The RT-qPCR was performed using 2 × Fas qPCR Master Mixture. The thermal cycling programme began with two minutes at 94 °C, followed by 40 cycles of 94 °C for 15 s and 60 °C for 30 s (primers used for RT-qPCR are listed in Table 2). The data were calculated using a 2^−ΔΔCt^ method, and the β-actin was used to normalise Marc-145 cells [35–38].

**Table 2:** **Primers for RT-qPCR amplification used in this study**

Gene	Sequence (5′–3′)	Size (bp)
PRRSV-N	F: ATAAGAAAAGCCCGGAGAAG	190
	R: TGCGTCGGCAAACTAAAC	
DDX3X	F: TTTGCTGGCCTAGACCTGAACTC	298
	R: CACCTCTGTCACCACGGC	
β-actin	F: AGGCCCAGAGCAAGAGAGG	463
	R: TCACGCACGATTTCCCGC	
GPX4	F: TTAGCCGCCTGTTCCGCC	211
	R: CATTGAGAGGCCACATTGGTG	
NCOA4	F: CTTCCAACTTCGTGGTGTCC	199
	R: GATCACAAACTGCTGGGAGG	
ACSL4	F CAAACCTGGAAGTCCATATCGC	186
	R: AACACCTTTCCATTTGGCTGC	
STEAP3	F: GGCTCTTCGTCTGCTTCTATG	359
	R: CTGCTTGACTGCCAGGTTG	

### Transcriptomic analysis

The Marc-145 cells were collected for RNA extraction using RNAiso Plus reagent (TaKaRa, Japan) under the manufacturer’s instructions. The mRNAs were enriched using magnetic beads with oligo (dT), and the mRNAs were sheared into fragments of approximately 200 bp by adding a fragmentation buffer. The double-stranded cDNAs were then synthesised using reverse transcriptase, end-repaired using End Repair Mix, and poly(A) tailed. Illumina HiSeq/MiSeq sequencing adapters were ligated to the cDNAs to construct a sequencing library. The raw reads underwent pre-processing to remove rRNA reads, sequencing adapters, short fragments, and other low-quality reads.

After genome mapping, Cufflinks v2.1.1 was run with a reference annotation to generate FPKM values for known gene models before identifying differentially expressed genes (DEGs) using Cuffdiff. Subsequently, the GO database was used to categorise the DEGs according to biological processes, cellular components, and molecular functions. Using the KEGG Orthology Based Annotation System (KOBAS), KEGG pathway enrichment analysis identified the most relevant biological pathways.

### Metabolome assay and data analysis

Marc-145 cells infected with PRRSV strain SD16 and *DDX3X* gene-silenced were pelleted and analysed by UHPLC (1290 Infinity LC, Agilent Technologies), coupled to a quadrupole time-of-flight (AB SCIEX Triple TOF 6600) analyser. The raw data from the mass spectrometry (MS) analysis was converted into MzXML files and processed for feature detection, retention time correction, and alignment using XCMS. The total ion chromatogram (TIC) of quality control (QC) samples was also compared with spectral overlap to identify variations caused by instrumental errors throughout the experiment. The results were evaluated through a range of statistical analyses, including univariate and multivariate analyses, differential metabolite screening, differential metabolite correlation analysis, and KEGG pathway analysis. Multivariate methods, such as OPLS-D, downscaled the collected multidimensional data while preserving the maximum original information. Such an analysis globally reflects inter-group differences as well as intra-group variability. Subsequent hierarchical clustering analysis (HCA) and metabolite correlation analysis allowed the study of the relationship between differentially expressed metabolites (DEMs). Finally, the KEGG pathway mapper function was used to identify the differential metabolic pathways.

### Metabolome and transcriptome association analysis

Subsequently, HCA based on Spearman correlation was performed to visualise the expression patterns of DEGs and DEMs. Overall, the close clustering of genes and metabolites indicated similar expression patterns and pointed towards their potential involvement in closely related biological processes or reaction steps. All DEGs and DEMs were also mapped to the KEGG pathway database to obtain their common pathway information. The main biochemical and signal transduction pathways were then analysed.

### Statistical analysis

Western blot and RT-qPCR experiments were each independently repeated in triplicate, and the statistical data analysis was conducted using GraphPad Prism 8.0 (GraphPad Software, Inc., San Diego, CA, USA). Each experiment in this study was repeated three times independently. As the data had a normal distribution (Shapiro–Wilk test; *p* > 0.05), the statistical differences between the different groups were compared using a two-way analysis of variance (ANOVA) (Tukey’s multiple comparisons test, with alpha = 0.05). The univariate statistical analysis (Fold Chang Analysis, t-test) and multivariate statistical methods (PCA, OPLS-da, OPLS) were integrated to analyse the DEGs and DEMs between different groups for the metabolome and/or transcriptome analysis. Additionally, Spearman’s nonparametric-correlation method was employed for correlation analysis.

## Results

### PRRSV infection up-regulates the expression of DDX3X

**Figure 1:**
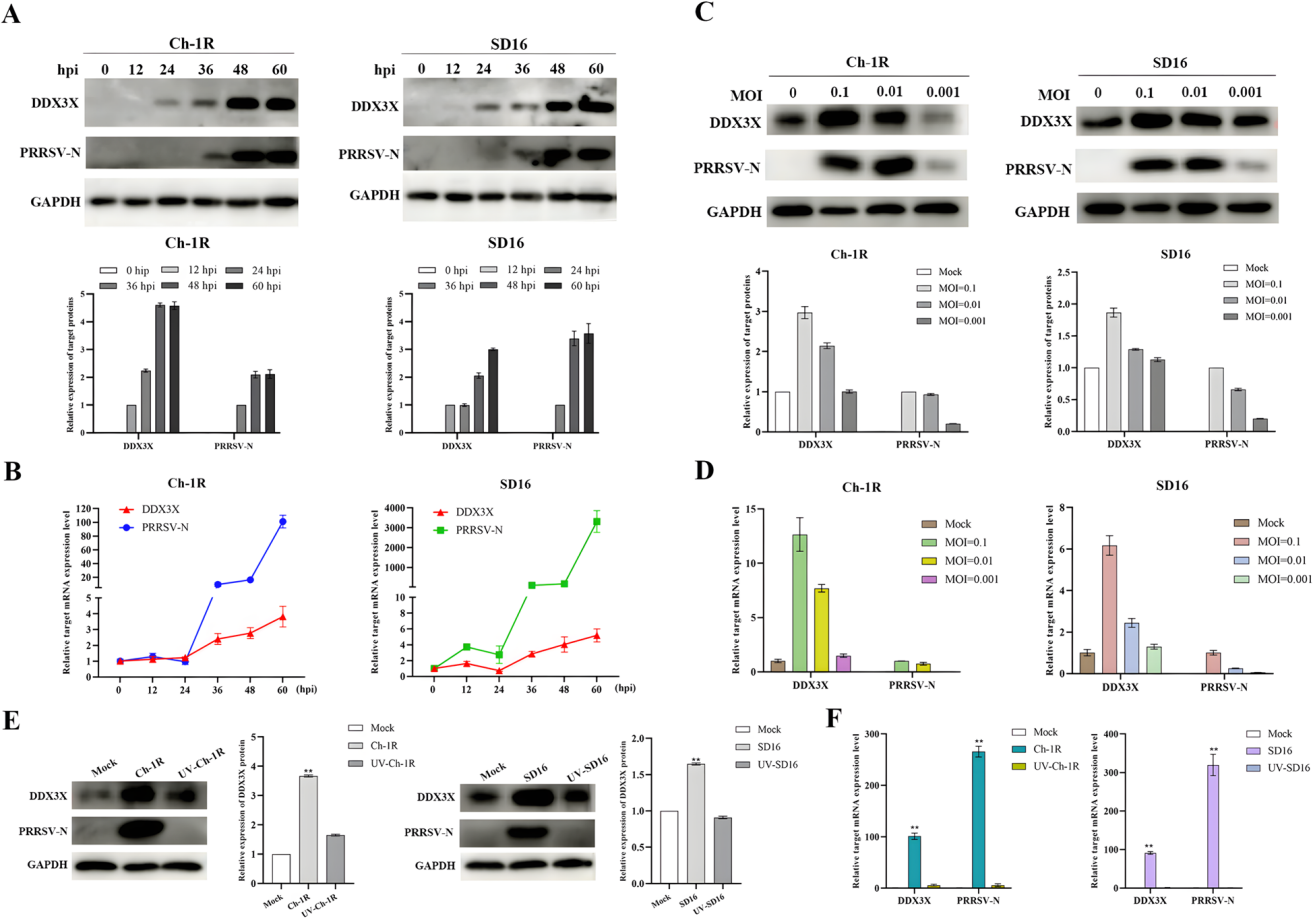
**mRNA and protein expression levels of DDX3X in PRRSV-infected Marc-145 cells.** **A**, **B** Western blot and RT-qPCR analysis of the expression of DDX3X in PRRSV-infected Marc-145 cells determined at different time points post-infection. **C**, **D** Western blot and RT-qPCR analysis of the expression of DDX3X in Marc-145 cells infected with varying doses of PRRSV. **E**, **F** Western blot and RT-qPCR analysis of the expression of DDX3X in Marc-145 cells infected with live or inactivated viruses. Each of the experiments was independently repeated in triplicate. Two-tailed Student’s t-test determined statistical analysis of comparisons, *n* = 3 ± SEM. The stars indicate significant differences (*P < 0.05 or **P < 0.001).

To investigate the effects of PRRSV infection on the in vitro expression of DDX3X, Marc-145 cells were infected with PRRSV strains Ch-1R and SD16, respectively. The levels of mRNA and protein expression for DDX3X increased significantly in virus-infected cells at different time points post-infection, along with the replication of both PRRSV Ch-1R and SD16 strains (Figure 1A and B) in a dose-dependent manner (Figure 1C and D). RT-qPCR and western blot assays analysed the expression levels of DDX3X. However, it was found that infectingMarc-145 cells with inactivated viruses did not affect the protein or mRNA expressions of DDX3X (Figure 1E and F). The results further indicated that the virus leads to an increase in DDX3X expression, which is dependent on both the duration of exposure and the amount of the virus. This enhanced expression is linked to the virus’s replication process.

### RK-33 inhibitor suppresses DDX3X expression and reduces PRRSV replication

**Figure 2:**
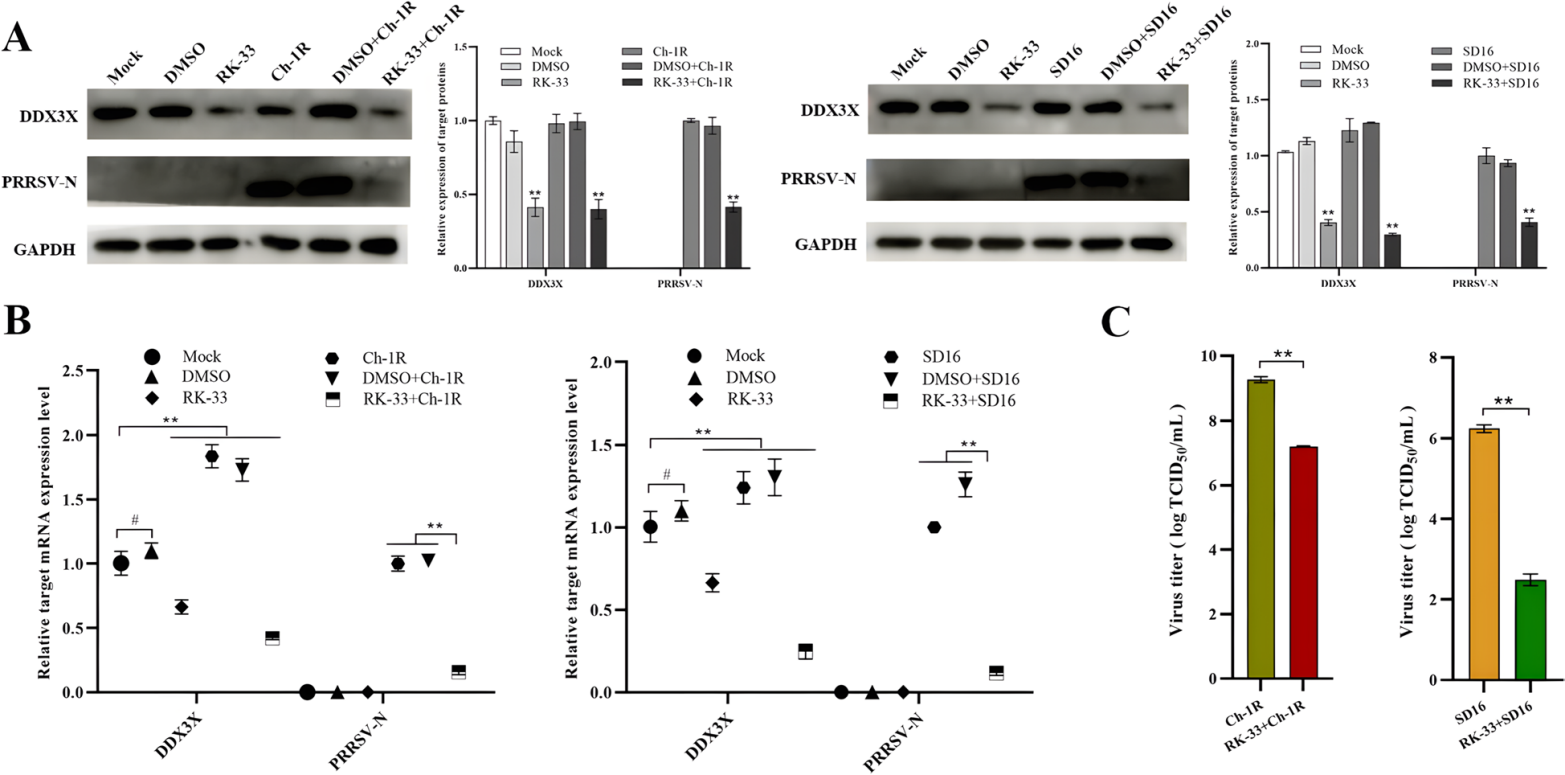
**Expression of DDX3X and virus proliferation in RK-33-treated and virus-infected Marc-145 cells**. **A**, **B** Western blot and RT-qPCR analysis of the expressions of DDX3X and PRRSV-N protein. **C** Virus titres of PRRS viruses in RK-33-treated and virus-infected Marc-145 cells were detected at 48 hpi. Two-tailed Student’s t-test determined statistical analysis of comparisons, *n* = 3 ± SEM. The stars indicate significant differences (*P < 0.05 or **P < 0.001).

The effect of DDX3X on PRRSV replication and proliferation was further examined by employing the chemical RK-33 inhibitor to suppress its expression. The optimal dose of RK-33 was determined to be 8.495 μM based on the IC_50_ of RK-33 in Marc-145 cells (Additional file 1). The later experiments showed that in RK-33-pretreated Marc-145 cells, there was a noteworthy decrease in the expression of DDX3X protein than in the mock or DMSO-treated cells (Figure 2A). The RK-33 pre-treatment cells showed a substantial reduction in PRRSV-N protein mRNA and protein expressions when infected with either SD16 or Ch-1R viruses (Figure 2A and B). TCID_50_ measurements showed that the viral titres of Ch-1R and SD16 strains significantly decreased in the group treated with RK-33 (Figure 2C). This outcome indicates that the suppression of DDX3X activity greatly reduced the production of PRRSV progenies.

### DDX3X promotes PRRSV replication at various stages of the viral replication

**Figure 3:**
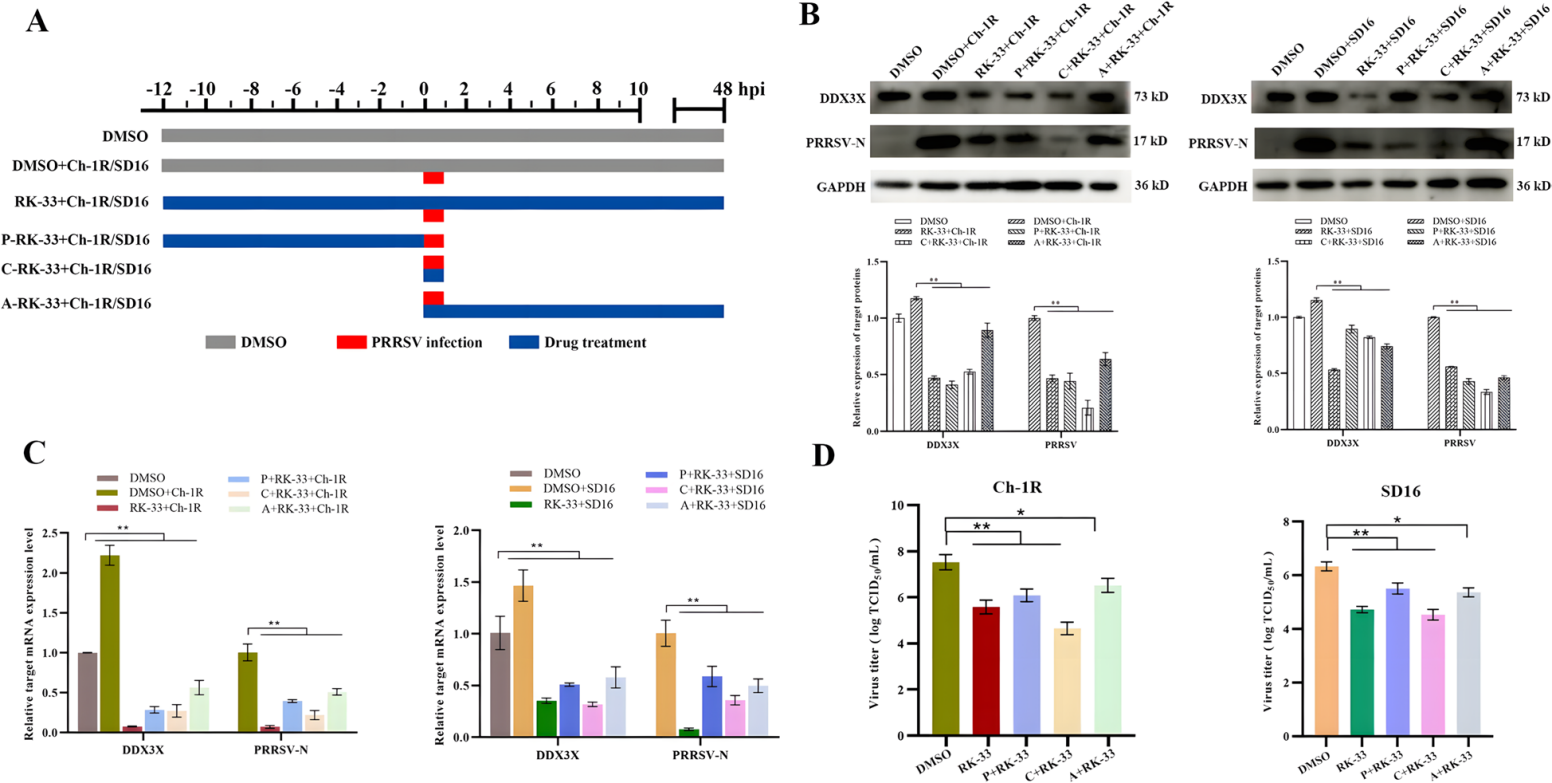
**Expression of DDX3X and virus replication in Marc-145 cells with distinct RK-33 treatments.** **A** Schematic of the time-of-addition experiment of RK-33 treatments. The Marc-145 cells were infected with PRRSV Ch-1R or SD16 at an MOI of 0.1 before being treated with RK-33: pre (− 12–0 h), during (0–1 h) and post (1–48 h) virus infection. DMSO was added at the same time as a mock control. **B**, **C** DDX3X and PRRSV-N protein expressions in Marc-145 cells treated with RK-33 at different time points and infected with PRRSV were determined at 48 hpi by western blotting and RT-qPCR. **D** Virus titres of PRRS viruses in Marc-145 cells treated with RK-33 were detected at 48 hpi. Two-tailed Student’s t-test determined statistical analysis of comparisons, *n* = 3 ± SEM. The stars indicate significant differences (*P < 0.05 or **P < 0.001).

Four groups of virus-infected cells were established to determine the role of DDX3X at distinct stages of PRRSV replication. In the first group (Figure 3A), the RK-33 inhibitor was present throughout all stages of virus replication (RK-33 + Ch-1R/SD16), whereas, in the second group, the cells were pre-treated with RK-33 for 12 h prior to viral infection (P + RK-33 + Ch-1R/SD16). In the third group, the RK-33 inhibitor was administered concurrently with the viral infection (C + RK-33 + Ch-1R/SD16), while in the final group, RK-33 inhibitors were administered after the virus had attached to the cells (A + RK-33 + Ch-1R/SD16). At 48 hpi, all cells were collected to extract proteins and RNA, which were then examined. The western blot analysis revealed that the levels of DDX3X and PRRSV-N proteins were lower in all four cases compared to the DMSO + Ch-1R/SD16 control (Figure 3B). This result was consistent with the findings of RT-qPCR (Figure 3C). TCID_50_ measurements indicate a significant decrease in viral titres across all four groups (Figure 3D). These findings suggest that reducing DDX3X activity effectively hinders all phases of viral replication, with a more pronounced effect observed during the initial stages of replication.

### Knockdown of DDX3X expression inhibits PRRSV replication

**Figure 4:**
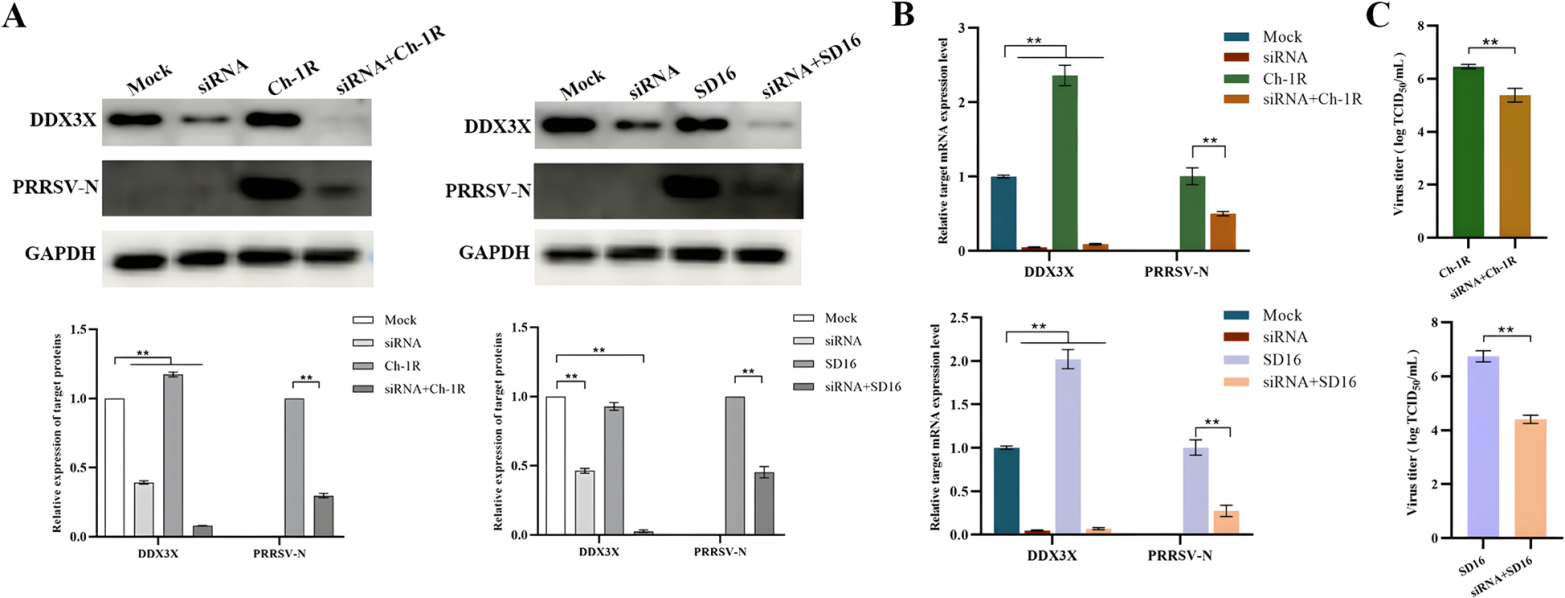
**Gene expression and virus proliferation in Marc-145 cells transfected with DDX3X-specific siRNA.** **A**, **B** DDX3X and PRRSV-N protein expressions in Marc-145 cells transfected with *DDX3X*-specific siRNA were analysed by western blot and RT-qPCR at 48 hpi. **C** Virus titres of PRRS viruses in Marc-145 cells transfected with *DDX3X*-specific siRNA. Two-tailed Student’s t-test determined statistical analysis of comparisons, *n* = 3 ± SEM. The stars indicate significant differences (*P < 0.05 or **P < 0.001).

Three specific siRNAs were synthesised and transfected into Marc-145 cells, all of which could considerably inhibit the expression of DDX3X at 20 pmol (Additional file 2). Before inoculation with Ch-1R or SD16 viruses, Marc-145 cells were transfected with siRNA1 for 12 h. The results indicated a reduction in N protein and ORF7 mRNA levels in cells transfected with siRNA at 48 h post-infection (Figure 4A and B). Subsequent TCID_50_ measurements revealed a substantial drop in PRRSV virus titres following siRNA-mediated silencing (Figure 4C). These findings demonstrate that the silencing of the *DDX3X* gene results in decreased replication and proliferation of PRRSV, which is consistent with earlier results.

### Overexpression of DDX3X promotes PRRSV replication

**Figure 5:**
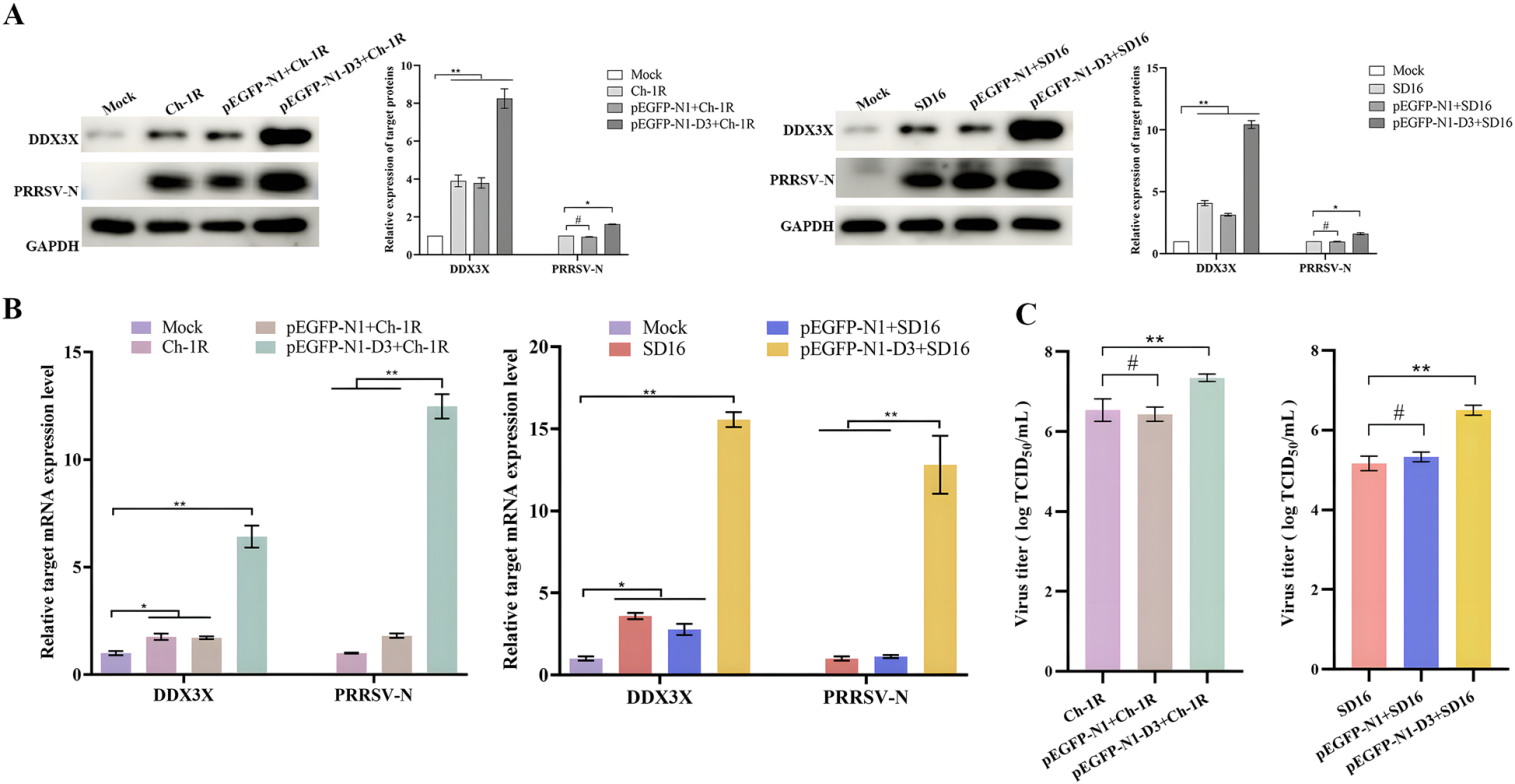
**Gene expression and virus proliferation in Marc-145 cells transfected with pEGFP-N1-DDX3X plasmid.** **A**, **B** Cells transfected with pEGFP-N1-DDX3X or empty pEGFP-N1 were infected with PRRSV before western blotting and RT-qPCR analysis. **C** Marc-145 cells were infected with PRRSV Ch-1R or SD16 12-h post-transfection with pEGFP-N1-DDX3X or empty pEGFP-N1. Supernatants were then collected for TCID_50_ measurement. Two-tailed Student’s t-test determined statistical analysis of comparisons, *n* = 3 ± SEM. The stars indicate significant differences (*P < 0.05 or **P < 0.001).

To provide further evidence of the positive regulatory role played by DDX3X in PRRSV replication, the pEGFP-N1-DDX3X plasmid was transfected into Marc-145 cells to augment the expression of DDX3X before infection with the virus. Evidence demonstrates that DDX3X was effectively up-regulated in Marc-145 cells (Additional file 3). Subsequent analyses showed that the elevated DDX3X expression discernibly increased both the mRNA and protein expression levels of the *PRRSV-N* gene (Figure 5A and B). Furthermore, virus titration showed that the overexpression of DDX3X led to a substantial increase in the proliferation of both Ch-1R and SD16 (Figure 5C). Therefore, it suggests that the excessive expression of DDX3X results in an amplification of virus generation.

### RNA-seq and data analyses

**Figure 6:**
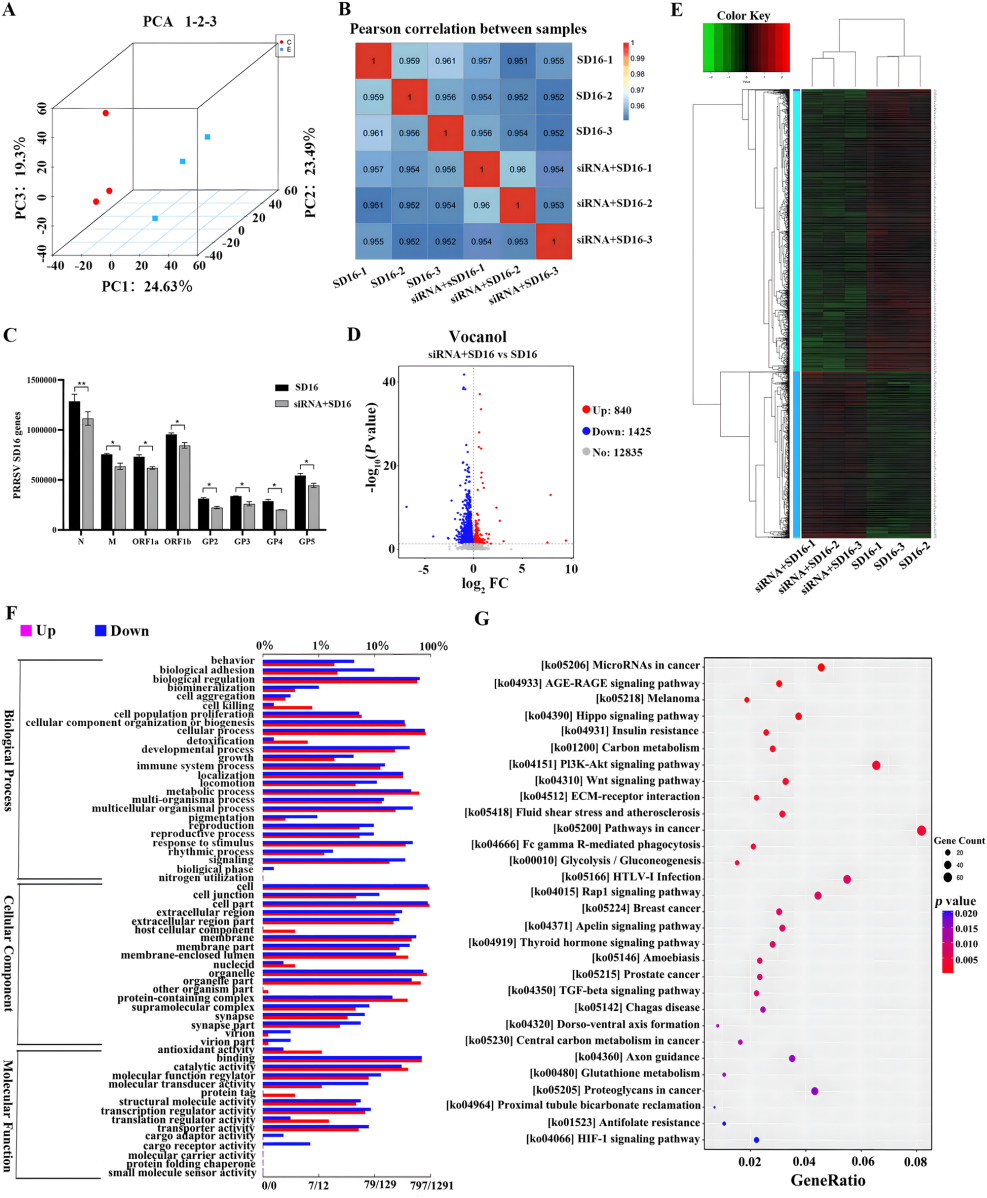
**Transcriptomics analysis of Marc-145 cells transfected with DDX3X-specific siRNA and infected with PRRSV.** **A** PCA plot of RNA-seq data. **B** Heatmap of correlation between samples from different groups. **C** Transcript levels of PRRSV N, M, ORF1a, ORF1b, GP2, GP3, GP4 and GP5 genes. **D** Volcanic map of the DEGs. **E** Heatmap of the DEGs. **F** GO terms of the DEGs. The lower x-axis represents the number of genes annotated to GO terms, while the upper x-axis represents the proportion of the total number of genes. Red lines represent up-regulated genes, and blue lines represent down-regulated ones. **G** KEGG enrichment bubble plot. The abscissa was the rate of the number of DEGs to the total number of DEGs, with the ordinate being the KEGG pathway. Two-tailed Student’s t-test determined statistical analysis of comparisons, *n* = 3 ± SEM. The stars indicate significant differences (*P < 0.05 or **P < 0.001).

To investigate the effects of DDX3X expression on the transcriptome of PRRSV-infected cells, RNA was extracted from the *DDX3X*-specific siRNA transfected or untransfected Marc-145 cells infected with SD16. This division was defined as the experimental group and control group, respectively. Principal component analysis (PCA) revealed a substantial separation between the two groups (Figure 6A), which was further evidenced by the sample correlation heatmap presenting a Pearson correlation coefficient of > 0.9 (Figure 6B). This finding implies both a good inter-group separation and excellent within-group repeatability and the successful establishment of a transcriptome model. The drop in the expression of the *N, M, ORF1a, ORF1b, GP2, GP3, GP4,* and *GP5* genes in SD16-infected cells compared with *DDX3X*-silenced and infected cells required observation (Figure 6C).

Furthermore, the volcano map revealed a total of 2265 DEGs. Out of these, 840 genes were found to be up-regulated, while 1425 genes were down-regulated (Figure 6D). Detailed information regarding these DEGs can be found in Additional files 4 and 5; the transcript level clustering of DGEs is shown in the heatmap of Figure 6E. Specifically, the majority of genes in the *DDX3X*-silenced and infected cells showed a decline compared to the control group. An analysis of GO annotation was conducted on the genes that were differentially expressed between the two groups, as illustrated in Figure 6F. The analysis revealed significant differences across multiple biological processes, which included biological regulation, cellular component organisation or biogenesis, cellular process, detoxification, developmental process, and immune system process. The analysis also revealed differences in cellular components, such as cell part, extracellular region part, membrane part, membrane-enclosed lumen, organelle part, and protein-containing complex.

Variations were found in molecular functions, including binding, catalytic activity, structural molecule activity, regulators of molecular function, transcriptional regulator activity, and transporter activity. The GO functional annotation for significant DEGs is presented in Additional file 6. KEGG pathway enrichment analysis was also performed (Figure 6G and Additional file 7), with the results revealing the pathways in cancer and the signalling pathways for P13K-Akt, Wnt and Rap1. The results suggest that the silencing of DDX3X may alter the proliferation, apoptosis, migration, and survival of SD16-infected cells. In brief, the RNA-seq data shows that the treatment with RK-33 has caused changes in the transcriptional landscape of both the host and the virus.

### Metabolomics analysis

**Figure 7:**
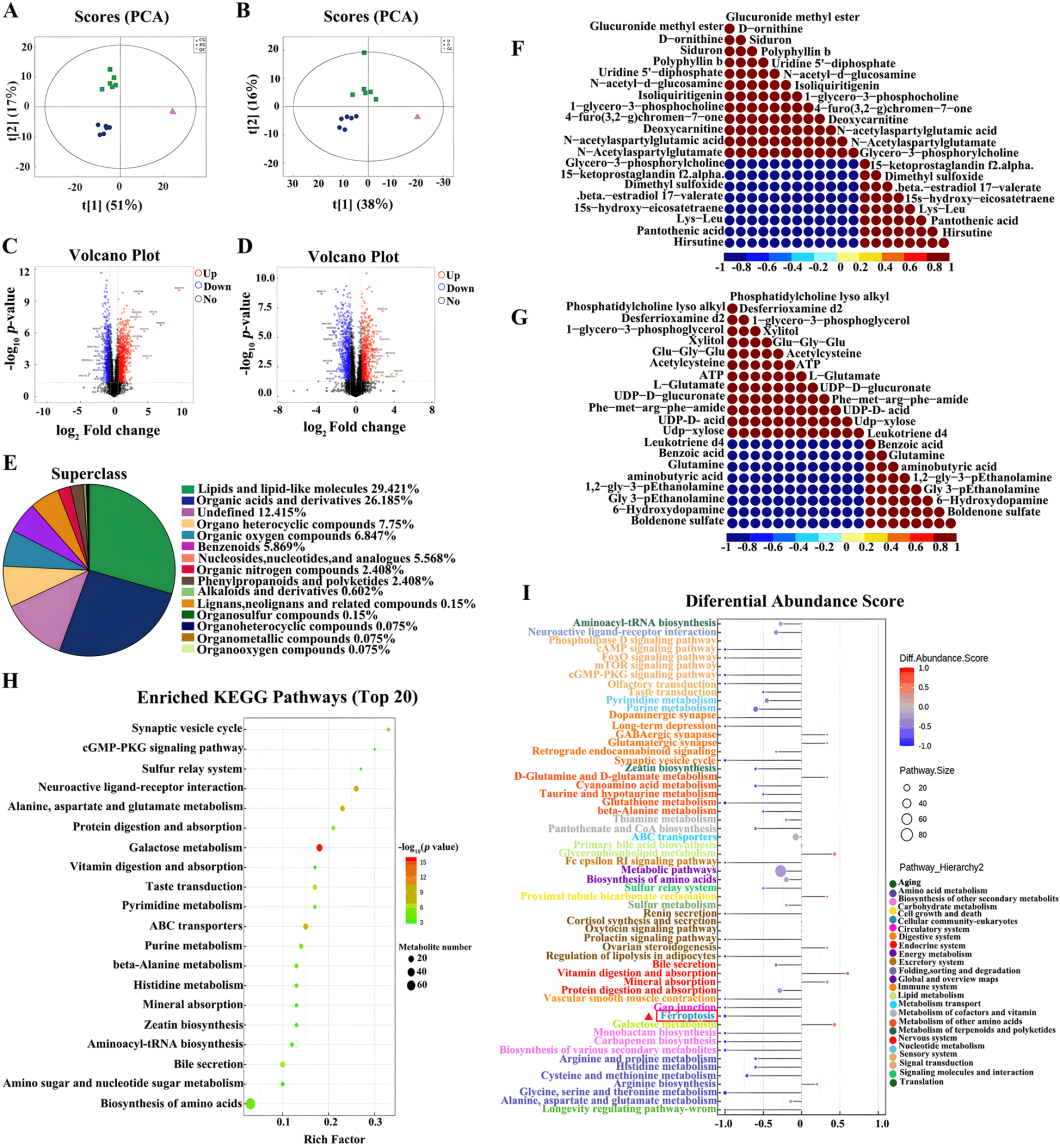
**Metabolomics of the PRRSV-infected Marc-145 cells transfected with DDX3X-specific siRNA.** **A**, **B** PCA plot in positive ion mode (left) and negative ion mode (right). **C**, **D** Volcanic diagram of DEMs in positive ion mode (left) and negative ion mode (right). **E** Percentage of DEMs by chemical classification. **F**, **G** Heatmap of correlations in positive ion mode (left) or negative ion mode (right). **H** Top 20 KEGG enrichment pathway bubble maps. The colour from green to red indicates the successively decreased *p*-values. **I** Differential abundance scores of the KEGG pathway are classified according to the hierarchy classification method.

In the metabolomics analysis of PRRSV-infected Marc-145 cells transfected with *DDX3X-*specific siRNA, Partial Least Squares Discriminant Analysis (PLS-DA) showed a clear separation of the QC samples from the control and experiment groups. The QC samples were closely clustered in positive and negative ion modes, as depicted in Figure 7A and B. These findings indicated that the experiment was reproducible, and the siRNA-based knockdown of DDX3X was the main factor responsible for the subsequent separation of samples on the PCA plot.

Volcano plots illustrate the DEMs (Figure 7C and D); the positive ion mode showed 248 noteworthy up-regulated DEMs and 162 down-regulated ones (Additional file 8). In the negative ion mode, 100 significant up-regulated DEMs were detected, accompanied by 132 significantly down-regulated DEMs (Additional file 9). All the identified metabolites were classified and counted based on their chemical classification information (Figure 7E). The results showed that the lipids and lipid-like molecules (29.421%), organic acids and derivatives (26.185%), and organic heterocyclic compounds (7.75%) were the major components. Furthermore, correlation analysis was conducted among important metabolites before visualising the results as a correlation heatmap (Figure 7F and G). A bubble plot was used to display the KEGG pathway enrichment analysis results. The plots indicated noticeable effects on the purine metabolism, ABC transport proteins, cGMP-PKG signalling pathway, and metabolic pathways (Figure 7H). The differential enrichment score plot for all enriched metabolic pathways is provided in Figure 7I. During this phase of the study, the AMP-activated protein kinase (AMPK) signalling pathway, ferroptosis, glutathione metabolism, and the metabolism of glycine, serine, and threonine all showed notable down-regulation. The KEGG pathway annotations for DEMs can be found in Additional file 10. Based on the available data, DDX3X has been shown to control cellular metabolic processes, primarily those associated with drug transport, metabolism, and immunological pathways.

### Integrated analysis of transcriptomics and metabolomics

**Figure 8:**
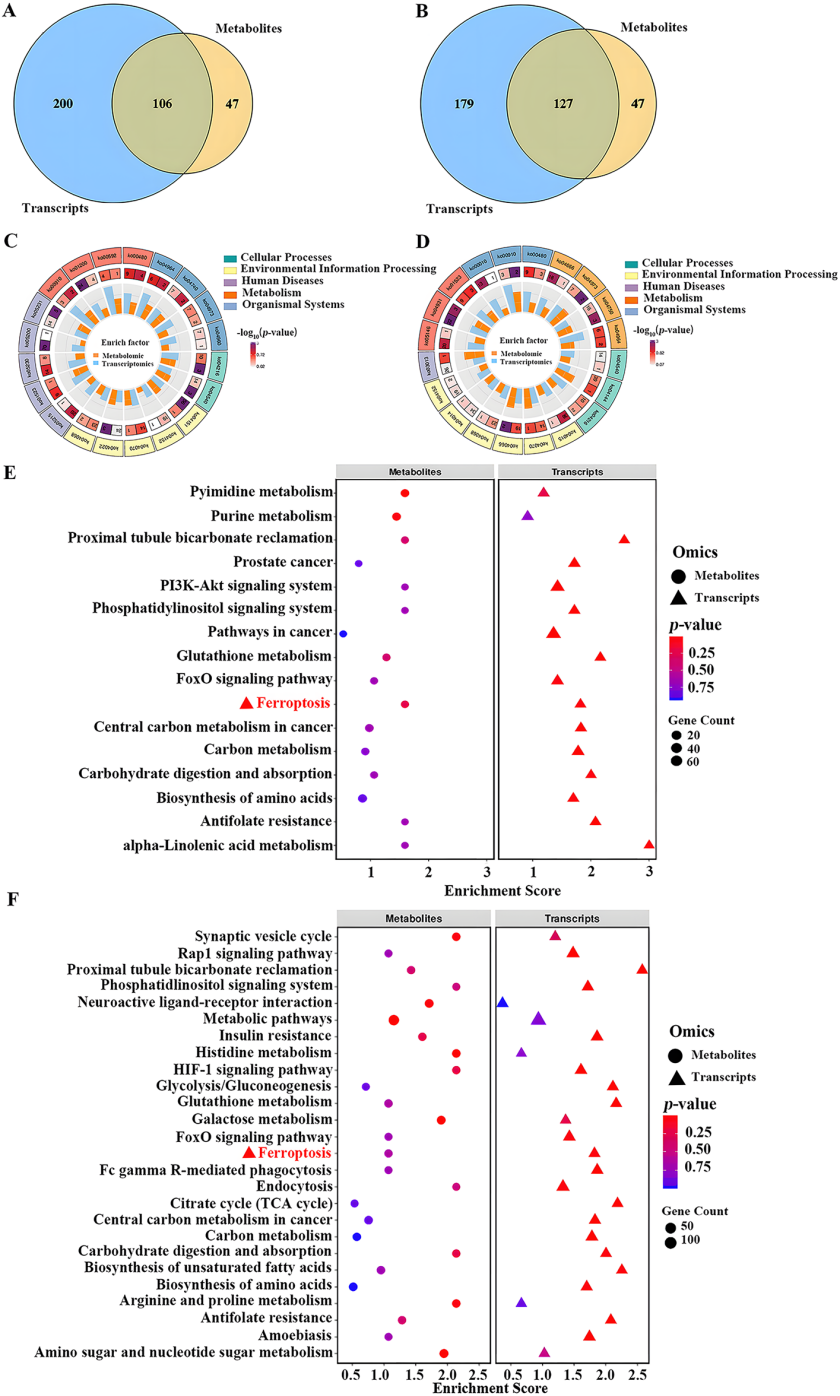
**The combinate analysis of metabolomics and transcriptomics.** **A**, **B** Venn diagrams showing the DMGs and DEMs of positive (left) or negative (right) ion modes in *DDX3X*-silenced cells compared with that in SD16-infected mock cells. **C**, **D** The top 20 pathways with the highest number of genes and metabolites in positive (left) or negative (right) ion modes. **E**, **F** Bubble diagram of the KEGG pathway enrichment analysis.

A Venn diagram was used to analyse the transcriptome and metabolome, identifying 106 important pathways in the positive ion mode and 127 pathways in the negative ion mode (Figure 8A and B). The top 20 co-expression pathways between DEGs and DEMs were mapped to uncover further the co-enriched pathways between the two groups (Figure 8C and D). Tables 3 and 4 list the first 20 pathways for positive and negative ions, respectively. The findings indicate that only six pathways or biological processes are involved in both positive and negative ions. These include ferroptosis, gap junction, the AMPK signalling pathway, the FoxO signalling pathway, proximal tubule bicarbonate reclamation, and carbohydrate digestion and absorption.

**Table 3:** **Pathway Top 20 for positive ions**

TermID	Description	Category
ko04216	Ferroptosis	Cellular processes
ko04540	Gap junction	Cellular processes
ko04151	PI3K-Akt signalling pathway	Environmental information processing
ko04152	AMPK signalling pathway	Environmental information processing
ko04070	Phosphatidylinositol signalling system	Environmental information processing
ko04022	cGMP-PKG signalling pathway	Environmental information processing
ko04068	FoxO signalling pathway	Environmental information processing
ko05215	Prostate cancer	Human diseases
ko01523	Antifolate resistance	Human diseases
ko05230	Central carbon metabolism in cancer	Human diseases
ko05200	Pathways in cancer	Human diseases
ko05231	Choline metabolism in cancer	Human diseases
ko00910	Nitrogen metabolism	Metabolism
ko01200	Carbon metabolism	Metabolism
ko00592	alpha-Linolenic acid metabolism	Metabolism
ko00480	Glutathione metabolism	Metabolism
ko04964	Proximal tubule bicarbonate reclamation	Organismal systems
ko04740	Olfactory transduction	Organismal systems
ko04973	Carbohydrate digestion and absorption	Organismal systems
ko04960	Aldosterone-regulated sodium reabsorption	Organismal systems

**Table 4:** **Pathway Top 20 for negative ions**

TermID	Description	Category
ko00480	Glutathione metabolism	Metabolism
ko04666	Fc gamma R-mediated phagocytosis	Organismal systems
ko04973	Carbohydrate digestion and absorption	Organismal systems
ko04750	Inflammatory mediator regulation of TRP channels	Organismal systems
ko04964	Proximal tubule bicarbonate reclamation	Organismal systems
ko04540	Gap junction	Cellular processes
ko04144	Endocytosis	Cellular processes
ko04216	Ferroptosis	Cellular processes
ko04015	Rap1 signalling pathway	Environmental information processing
ko04070	Phosphatidylinositol signalling system	Environmental information processing
ko04066	HIF-1 signalling pathway	Environmental information processing
ko04068	FoxO signalling pathway	Environmental information processing
ko04014	Ras signalling pathway	Environmental information processing
ko04152	AMPK signalling pathway	Environmental information processing
ko03013	RNA transport	Genetic information processing
ko05146	Amoebiasis	Human diseases
ko04931	Insulin resistance	Human diseases
ko01523	Antifolate resistance	Human diseases
ko00010	Glycolysis / Gluconeogenesis	Metabolism
ko00910	Nitrogen metabolism	Metabolism

Furthermore, after conducting an enrichment function analysis of the KEGG pathway, it was discovered that ferroptosis, the FoxO signalling pathway, the carbohydrate digestion and absorption pathway, glutathione metabolism, and amino acid biosynthesis were all associated with both positive and negative particles (Figure 8E and F). These targeted molecules and signalling pathways were mainly involved in cell growth, death, and material metabolism. Detailed information on the positive or negative ion results of DEGs, DEMs and KEGG enrichment pathways is shown in Additional files 11 and 12, respectively.

### Silencing DDX3X inhibits PRRSV-induced ferroptosis

**Figure 9:**
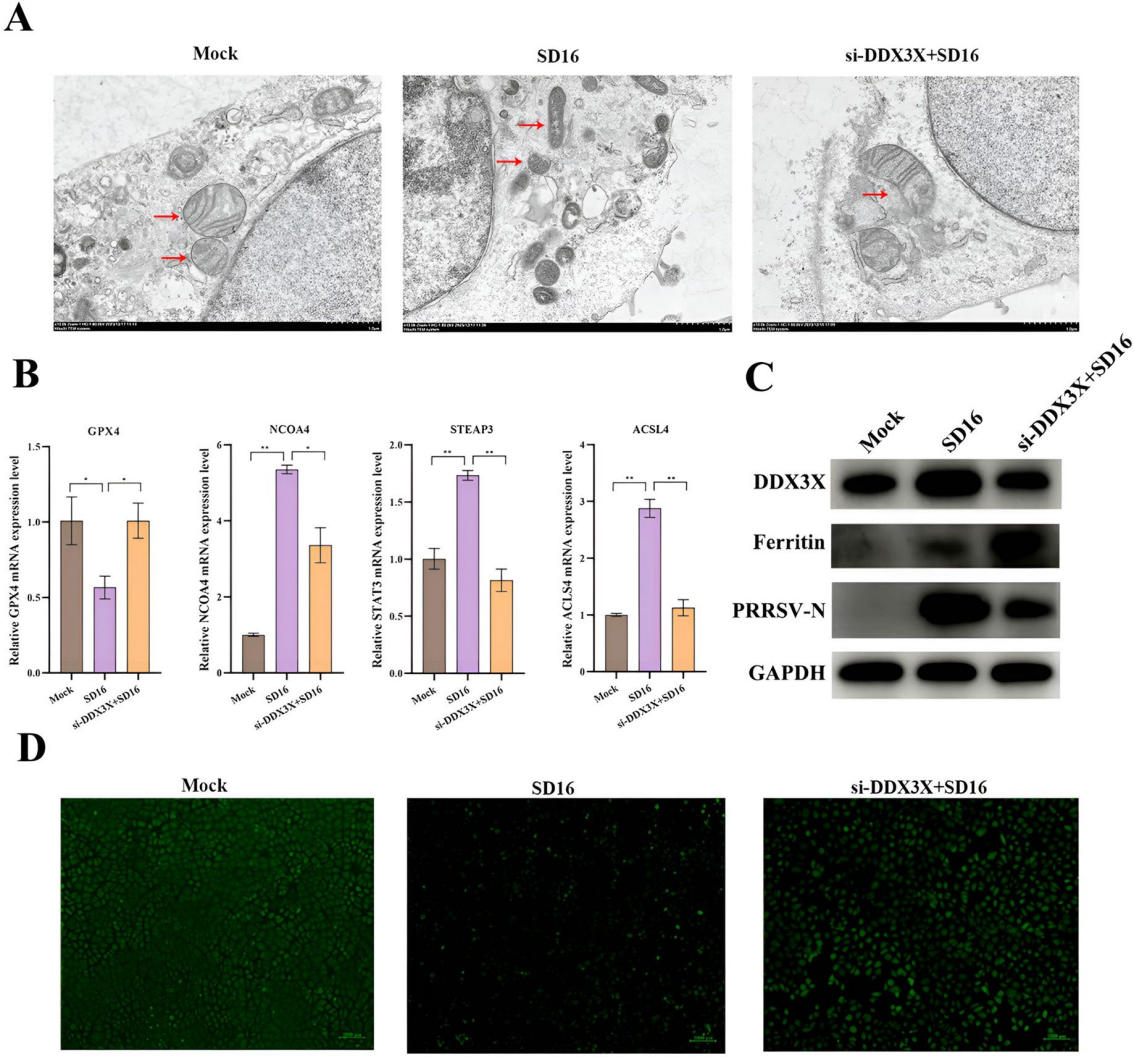
**PRRSV-induced ferroptosis in DDX3X-silenced Marc-145 cells.** **A** Electron microscopy image of mitochondria in Marc-145 cells. **B** Relative expression levels of ferroptosis-related genes *GPX4*, *ACST4, SCOA4,* and *STEAP3*. **C** Western blot analysis of ferritin. **D** Calcein-AM assay for the LIP quantification in mock control, SD16-infected or siRNA-transfected cells. Two-tailed Student’s t-test determined statistical analysis of comparisons, *n* = 3 ± SEM. The stars indicate significant differences (*P < 0.05 or **P < 0.001).

To examine the effect of DDX3X on ferroptosis triggered by PRRSV infection, DDX3X in Marc-145 cells was initially suppressed before exposing the cells to the SD16 virus. TEM imaging revealed the characteristic morphological features of ferroptosis in SD16-infected mock cells, including mitochondrial shrinkage, decrease or absence of mitochondrial cristae, and cytoplasmic membrane rupture (Figure 9A).

In contrast, the mitochondrial morphology in the *DDX3X*-silenced cells was normal (Figure 9B). A marked down-regulation of *GPX4* mRNA was observed in SD16-infected mock cells, while in the *DDX3X*-silenced cells with virus infection, expression of *GPX4* was considerably up-regulated. However, in the virus-infected mock cells, the mRNA levels of *NCOA4*, *ACSL4*, and *STEAP3* considerably increased while being down-regulated in the *DDX3X*-silenced cells with virus infection (Figure 9C). The western blot analysis revealed an elevated ferritin accumulation in the cells infected with the virus and silenced for *DDX3X*, suggesting that the reduced expression of *NCOA4* may hinder the process of ferritin autophagy (Figure 9D). Labile iron pool (LIP) was measured using the Calcein-AM assay, and a substantial decrease in fluorescence occurred in SD16-infected mock cells. Conversely, a sizeable increase in fluorescence was observed in the *DDX3X*-silenced and virus-infected cells (Figure 9E). This finding suggests that SD16 infection induces the accumulation of Fe^2+^ considerably and thus results in cellular ferroptosis, while the silence of DDX3X reduces ferroptosis by lowering Fe^2+^.

### Erastin-induced ferroptosis promotes PRRSV replication

**Figure 10:**
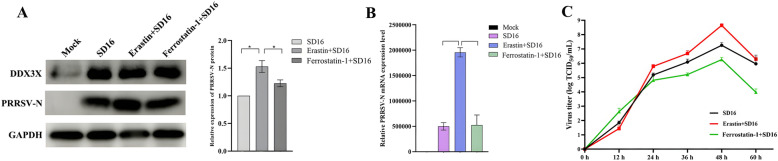
**Virus growth curve and gene expression levels in the Erastin or Ferrostatin-1-treated Marc-145 cells.** **A**, **B** Western blot and RT-qPCR assays analysed gene expression levels in Erastin or Ferrostatin-1-treated Marc-145 cells. **C** Virus growth curves of PRRSV in Erastin or Ferrostatin-1-treated Marc-145 cells determined by TCID_50_ titration. Two-tailed Student’s t-test determined statistical analysis of comparisons, *n* = 3 ± SEM. The stars indicate significant differences (*P < 0.05 or **P < 0.001).

To evaluate the influence of ferroptosis on PRRSV replication, Marc-145 cells were initially exposed to Erastin or Ferrostatin-1, followed by infection with SD16. Erastin therapy resulted in a substantial up-regulation of PRRSV-N protein expression, as demonstrated by western blot and RT-qPCR analysis (Figure 10A and B). However, the mRNA expression of the PRRSV-N protein was unchanged in Ferrostatin-1-treated cells. The virus proliferation curves also showed a noteworthy increase of PRRSV in Erastin-treated cells, while in Ferrostatin-1-treated cells, it was decreased (Figure 10C).

## Discussion

Previous studies have shown that several RNA viruses rely on DDX3X to enhance their replication. For instance, snakehead vesiculovirus (SHVV) phosphoproteins hijacked DDX3X to facilitate virus replication via phosphoprotein stabilisation [39]. The human immunodeficiency virus (HIV) escapes host immune surveillance by blocking the DDX3X-MAV pathway to promote self-replication [40]. Furthermore, it has been demonstrated that the peptides produced from the HCV core protein may inhibit the genotype 1b viral genome replication by interacting with DDX3X [41]. In addition, the vesicular stomatitis virus (VSV) and Sendai virus (SeV) achieve immune escape by inducing RNF39 expression to promote the ubiquitinated degradation of DDX3X [42]. The study found that both strains of PRRSV, Ch-1R and SD16, can lead to an increase in DDX3X expression. This increase is directly linked to viral replication and depends on the duration of exposure and the amount of virus present.

Conversely, virus replication was decreased when DDX3X expression was reduced, or its activity inhibited using siRNA-induced gene silencing or the specific inhibitor RK-33. The overexpression of DDX3X has been shown to promote virus replication considerably. Based on the data, it seems likely that DDX3X plays a potentially crucial role in regulating PRRSV replication, particularly in the early stages. The DDX3X protein has also been discovered to have antiviral effects on the dengue virus (DENV). It is important to note that only the N-terminus of DDX3X is crucial for its ability to resist virus infection [43], which indicates that DDX3X has many critical functions in the biology of several viruses, which requires further investigation.

This study used a combination of transcriptomics and metabolomics analysis to investigate the biological pathways influenced by DDX3X in relation to PRRSV replication. The objective was to identify the key differential signalling pathways and biological processes associated with PRRSV infection. Transcriptomics data showed 2265 DEGs, of which there are 840 up-regulated and 1425 down-regulated genes. In addition, several PRRSV genes were also found to be down-regulated in *DDX3X*-silenced cells. Based on the gene functions determined by GO enrichment analysis, the DEGs were mainly involved in biological processes, such as multicellular and developmental processes. The analysis suggests that DDX3X may regulate the virus-infected cells to actively regulate physiological states, including cell cycle, apoptosis, and metabolic pathways. Additionally, the study found that DDX3X can affect the replication of PRRSV by regulating ferroptosis.

Furthermore, suppressing the *DDX3X* gene enabled the host cell to specifically react and control the surrounding environment, particularly through immunological response and neuromodulation. KEGG pathway analysis further showed that the DEGs were mainly involved in pathways related to cancer, as well as in the P13K-Akt, Wnt, and Rap1 signalling pathways, which participate and contribute to multiple processes of cell survival, proliferation, differentiation, and/or the progress of the disease [44–46]. For the metabolomic analysis, 410 DEMs were detected between PRRSV-infected cells and *DDX3X*-silenced infected cells in the positive ion mode. In the negative ion mode, 232 DEMs were identified. The differential enrichment score plot results for all enriched metabolic pathways indicate a significant down-regulation of several pathways, including the cAMP signalling pathway, glutathione metabolism, ferroptosis, and glycine, serine, and threonine metabolism. In addition, many immune-related pathways, such as the Fc epsilon RI signalling pathway, the cGMP-PKG signalling pathway, the cAMP signalling pathway, and platelet activation, were also found to be differentially involved in PRRSV infection. Overall, this suggests that DDX3X can control a variety of PRRSV-induced gene expression, metabolite levels, and many physiologic processes within host cells.

Both transcriptomics and metabolomics analyses suggest that DEGs and DEMs in *DDX3X*-silenced and PRRSV-infected cells are likely associated with the ferroptosis pathway. Ferroptosis is a type of cell death regulated by iron dependency [47]. It affects glutathione peroxidase, either directly or indirectly, through many routes. As such, this effect reduces the cell’s ability to fight against oxidative stress, increases ROS and lipid peroxidation accumulation, and, ultimately, programmed cell death. In this study, SD16-infected cells showed mitochondrial density, disrupt, and shrinkage.

Conversely, mitochondrial morphology was unchanged in DDX3X-inhibited cells. The mRNA of ferroptosis factors (*NCOA4, ACSL4,* and *STEAP3*), ferritin, and Fe^2+^ expression levels were substantially up-regulated to promote ferroptosis in SD16-infected cells. However, silencing of DDX3X reduced ferroptosis through the above indicators. Subsequently, Erastin and Ferrostatin-1 were used to promote and inhibit ferroptosis in SD16-infected cells, with the results showing that ferroptosis promotes PRRSV infection. These data suggest that PRRSV infection induces ferroptosis and that DDX3X may promote PRRSV replication by facilitating ferroptosis.

An integrated analysis of transcriptomics and metabolomics data revealed that the expression levels of *NCOA4*, *STEAP3*, and *ACSL4* genes, as well as the metabolites L-Cystine, L-Cysteine, and DL-Glutamic, were substantially reduced in the *DDX3X*-silenced cells. On the other hand, the expression levels of *GPX4* and *GSS* genes increased considerably. These results indicate that the intracellular reduction of Cystine to Cysteine functions in biological processes such as glutathione (GSH) and protein synthesis [48]. The *GPX4* can protect cells from the threat of ferroptosis in a GSH-dependent manner [49, 50]. The deficiency of cyst(e)ine, cystine, or cysteine inhibits GSH synthesis and thus affects *GPX4* function, inducing ferroptosis [51].

**Figure 11:**
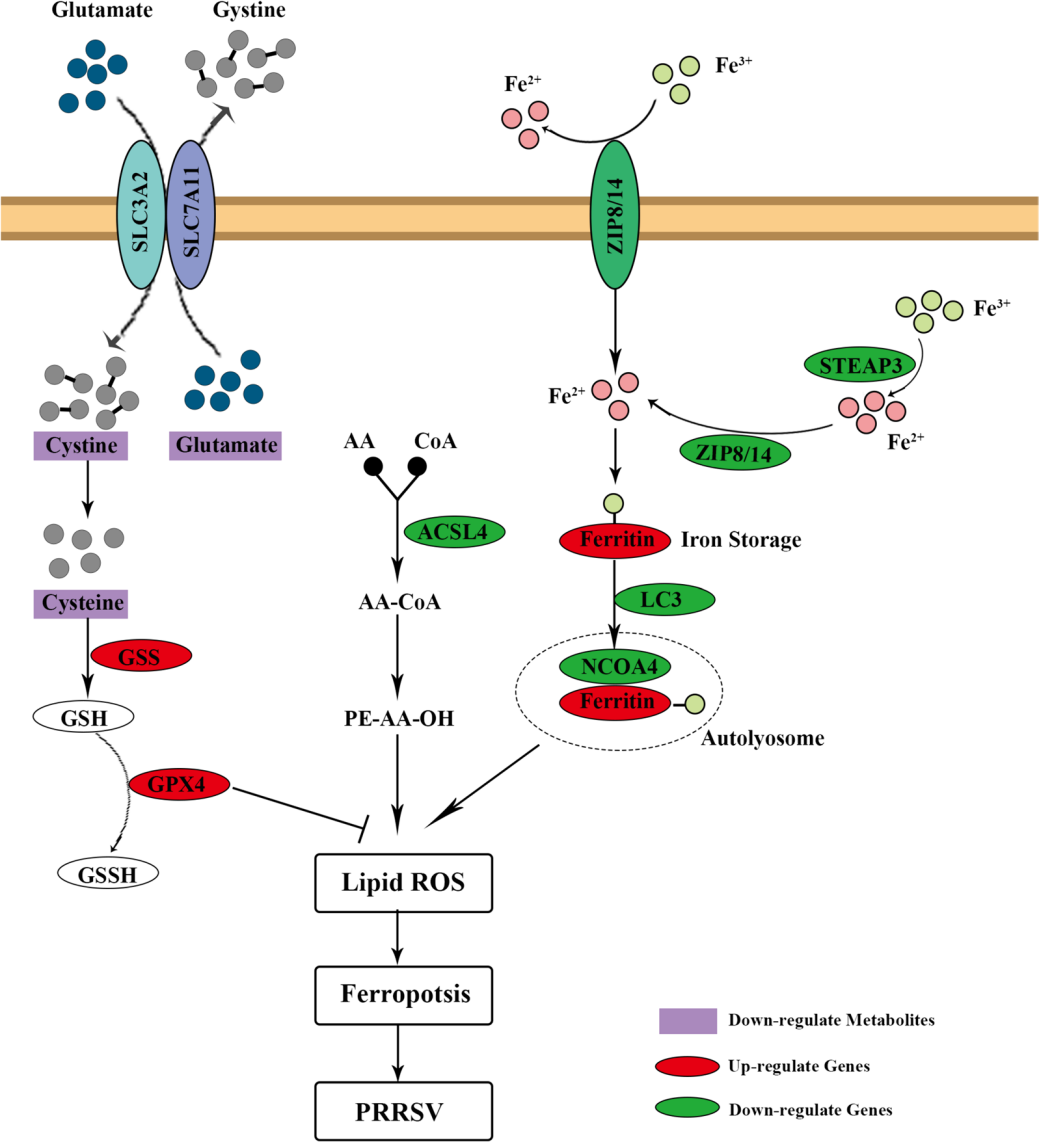
**Schematic of the regulatory mechanism of DDX3X-mediated ferroptosis in PRRSV-infected cells**.

Recently, it has been established that Cyst(e)ine deficiency can induce ferroptosis by inhibiting the Rag-mTORC1-4EBPs signalling pathway and its mediated translational expression [52]. However, the decrease in Cyst(e)ine levels in the cells where *DDX3X* was silenced did not affect the expression of *GPX4* in the current study. This outcome suggests that alternative pathways might control the expression of *GPX4* to prevent ferroptosis. For *STEAP3*, it converts Fe^3+^ into Fe^2+^, and the latter is released to the unstable iron pool in the cytoplasm to induce ferroptosis [53]. Previous works have recognised *NCOA4*-mediated ferritinophagy as an indispensable process for releasing free iron into the LIP. This study also found that inhibition of DDX3X down-regulated the expression of *NCOA4*, further alleviating intracellular free iron levels and ferritin accumulation. These results signify that DDX3X-induced ferroptosis is closely related to *NCOA4*-mediated ferritin degradation. These data suggest that, as demonstrated in Figure 11, DDX3X may induce ferroptosis through multiple pathways, such as System Xc- and Ferritinophagy, to promote the replication of PRRSV.

Interestingly, it has been shown that the severe acute respiratory syndrome coronavirus 2 (SARS-CoV-2) induces low levels of GSH and decreases the expression of *GPX4* to induce the occurrence of ferroptosis [47]. The other RNA virus, Newcastle disease virus (NDV), may induce ferroptosis through nutrient deprivation and ferritinophagy [54]. The outcome of this is that more viruses may potentially benefit from ferroptosis. In the future, the host cellular and viral proteins that can interact with DDX3X and participate in virally-induced ferroptosis deserve to be further studied.

### Supplementary Information

**Additional file 1. IC**_**50**_**. **Marc-145 cells were treated with various concentrations of RK-33, and cell viability was determined using the CCK-8 assay.

**Additional file 2. Evaluation of the effect of siRNA interference.** Marc-145 cells, transfected with siRNA-DDX3X or siRNA-negative control (NC), were harvested and analysed by western blotting (A) and RT-qPCR (B).

**Additional file 3. Validation of pEGFP-N1-DDX3X overexpression.** Green fluorescence indicates successful transfection of DDX3X overexpression plasmid and control plasmid.

**Additional file 4. Detailed information on up-regulated DEGs.**

**Additional file 5. Detailed information on down-regulated DEGs.**

**Additional file 6. GO functional annotation of DEGs.**

**Additional file 7. KEGG pathway enrichment analysis of DEGs.**

**Additional file 8. Detailed information on DEMs identified in positive ion mode.**

**Additional file 9. Detailed information on DEMs identified in negative ion mode.**

**Additional file 10. KEGG pathway enrichment analysis of DEMs.**

**Additional file 11. KEGG pathway analysed by combined DEGs and DEMs in positive ion mode.**

**Additional file 12. KEGG pathway analysed by combined DEGs and DEMs in negative ion mode.**

## Data Availability

All data generated or analysed during this study are included in this published article.

## References

[1] Albina E (1997) Epidemiology of porcine reproductive and respiratory syndrome (PRRS): an overview. Vet Microbiol 55:309–316 9220627 10.1016/S0378-1135(96)01322-3

[2] Whitworth KM, Rowland RR, Ewen CL, Trible BR, Kerrigan MA, Cino-Ozuna AG, Samuel MS, Lightner JE, McLaren DG, Mileham AJ, Wells KD, Prather RS (2016) Gene-edited pigs are protected from porcine reproductive and respiratory syndrome virus. Nat Biotechnol 34:20–22 26641533 10.1038/nbt.3434

[3] Murtaugh MP, Elam MR, Kakach LT (1995) Comparison of the structural protein coding sequences of the VR-2332 and Lelystad virus strains of the PRRS virus. Adv Virol 140:1451–1460 10.1007/BF01322671 PMC7086642 7661696

[4] Chand RJ, Trible BR, Rowland RR (2012) Pathogenesis of porcine reproductive and respiratory syndrome virus. Curr Opin Virol 2:256–263 22709514 10.1016/j.coviro.2012.02.002

[5] Jusa ER, Inaba Y, Kouno M, Hirose O (1997) Effect of heparin on infection of cells by porcine reproductive and respiratory syndrome virus. Am J Vet Res 55:488–491 10.2460/ajvr.1997.58.05.488 9140556

[6] Nelsen CJ, Murtaugh MP, Faaberg KS (1999) Porcine reproductive and respiratory syndrome virus comparison: divergent evolution on two continents. J Virol 73:270–280 9847330 10.1128/JVI.73.1.270-280.1999 PMC103831

[7] Garcia-Sastre A (2010) Influenza virus receptor specificity: disease and transmission. Am J Pathol 176:1584–1585 20203283 10.2353/ajpath.2010.100066 PMC2843447

[8] Valicek L, Psikal I, Smid B, Rodak L, Kubalikova R, Kosinova E (1997) Isolation and identification of porcine reproductive and respiratory syndrome virus in cell cultures. Vet Med 42:281–287 9416008

[9] Wang J, Lin S, Quan D, Wang H, Huang J, Wang Y, Ren T, Ouyang K, Chen Y, Huang W, Luo T, Wei Z (2020) Full genomic analysis of new variants of porcine reproductive and respiratory syndrome virus revealed multiple recombination events between different lineages and sublineages. Front Vet Sci 7:603 33134336 10.3389/fvets.2020.00603 PMC7511543

[10] Allende R, Laegreid WW, Kutish GF, Galeota JA, Wills RW, Osorio FA (2000) Porcine reproductive and respiratory syndrome virus: description of persistence in individual pigs upon experimental infection. J Virol 74:10834–10837 11044133 10.1128/JVI.74.22.10834-10837.2000 PMC110963

[11] Rowland RR, Lawson S, Rossow K, Benfield DA (2003) Lymphoid tissue tropism of porcine reproductive and respiratory syndrome virus replication during persistent infection of pigs originally exposed to virus in utero. Vet Microbiol 96:219–235 14559170 10.1016/j.vetmic.2003.07.006 PMC7172578

[12] Chaudhari J, Liew CS, Workman AM, Riethoven JM, Steffen D, Sillman S, Vu HLX (2020) Host transcriptional response to persistent infection with a live-attenuated porcine reproductive and respiratory syndrome virus strain. Viruses 12:817 32731586 10.3390/v12080817 PMC7474429

[13] Yoon KJ, Wu LL, Zimmerman JJ, Hill HT, Platt KB (1996) Antibody-dependent enhancement (ADE) of porcine reproductive and respiratory syndrome virus (PRRSV) infection in pigs. Viral Immunol 9:51–63 8733920 10.1089/vim.1996.9.51

[14] Yoon KJ, Wu LL, Zimmerman JJ, Platt KB (1997) Field isolates of porcine reproductive and respiratory syndrome virus (PRRSV) vary in their susceptibility to antibody dependent enhancement (ADE) of infection. Vet Microbiol 55:277–287 9220623 10.1016/S0378-1135(96)01338-7

[15] Shi P, Su Y, Li Y, Zhang L, Lu D, Li R, Zhang L, Huang J (2019) The alternatively spliced porcine FcgammaRI regulated PRRSV-ADE infection and proinflammatory cytokine production. Dev Comp Immunol 90:186–198 30273630 10.1016/j.dci.2018.09.019

[16] Wan B, Chen X, Li Y, Pang M, Chen H, Nie X, Pan Y, Qiao S, Bao D (2019) Porcine FcgammaRIIb mediated PRRSV ADE infection through inhibiting IFN-beta by cytoplasmic inhibitory signal transduction. Int J Biol Macromol 138:198–206 31284005 10.1016/j.ijbiomac.2019.07.005

[17] Kappes MA, Faaberg KS (2015) PRRSV structure, replication and recombination: origin of phenotype and genotype diversity. Virology 479–480:475–486 25759097 10.1016/j.virol.2015.02.012 PMC7111637

[18] Ariumi Y (2014) Multiple functions of DDX3 RNA helicase in gene regulation, tumorigenesis, and viral infection. Front Genet 5:423 25538732 10.3389/fgene.2014.00423 PMC4257086

[19] Valiente-Echeverria F, Hermoso MA, Soto-Rifo R (2015) RNA helicase DDX3: at the crossroad of viral replication and antiviral immunity. Rev Med Virol 25:286–299 26174373 10.1002/rmv.1845

[20] Samir P, Kesavardhana S, Patmore DM, Gingras S, Malireddi RKS, Karki R, Guy CS, Briard B, Place DE, Bhattacharya A, Sharma BR, Nourse A, King SV, Pitre A, Burton AR, Pelletier S, Gilbertson RJ, Kanneganti TD (2019) DDX3X acts as a live-or-die checkpoint in stressed cells by regulating NLRP3 inflammasome. Nature 573:590–594 31511697 10.1038/s41586-019-1551-2 PMC6980284

[21] Soto-Rifo R, Ohlmann T (2013) The role of the DEAD-box RNA helicase DDX3 in mRNA metabolism. Wiley Interdiscip Rev RNA 4:369–385 23606618 10.1002/wrna.1165

[22] Chahar HS, Chen S, Manjunath N (2013) P-body components LSM1, GW182, DDX3, DDX6 and XRN1 are recruited to WNV replication sites and positively regulate viral replication. Virology 436:1–7 23102969 10.1016/j.virol.2012.09.041 PMC3545066

[23] Li C, Ge LL, Li PP, Wang Y, Dai JJ, Sun MX, Huang L, Shen ZQ, Hu XC, Ishag H, Mao X (2014) Cellular DDX3 regulates Japanese encephalitis virus replication by interacting with viral un-translated regions. Virology 449:70–81 24418539 10.1016/j.virol.2013.11.008 PMC7111930

[24] Oda S, Schroder M, Khan AR (2009) Structural basis for targeting of human RNA helicase DDX3 by poxvirus protein K7. Structure 17:1528–1537 19913487 10.1016/j.str.2009.09.005

[25] Ariumi Y, Kuroki M, Abe K, Dansako H, Ikeda M, Wakita T, Kato N (2007) DDX3 DEAD-box RNA helicase is required for hepatitis C virus RNA replication. J Virol 81:13922–13926 17855521 10.1128/JVI.01517-07 PMC2168844

[26] Sun C, Pager CT, Luo G, Sarnow P, Cate JH (2010) Hepatitis C virus core-derived peptides inhibit genotype 1b viral genome replication via interaction with DDX3X. PLoS ONE 5:e12826 20862261 10.1371/journal.pone.0012826 PMC2941470

[27] Wang H, Ryu WS (2010) Hepatitis B virus polymerase blocks pattern recognition receptor signaling via interaction with DDX3: implications for immune evasion. PLoS Pathog 6:e1000986 20657822 10.1371/journal.ppat.1000986 PMC2904777

[28] Heaton SM, Atkinson SC, Sweeney MN, Yang SNY, Jans DA, Borg NA (2019) Exportin-1-dependent nuclear export of DEAD-box helicase DDX3X is central to its role in antiviral immunity. Cells 8:1181 31575075 10.3390/cells8101181 PMC6848931

[29] Niu Q, Cheng Y, Wang H, Yan Y, Sun J (2019) Chicken DDX3X activates IFN-beta via the chSTING-chIRF7-IFN-beta signaling axis. Front Immunol 10:822 31057547 10.3389/fimmu.2019.00822 PMC6478769

[30] Breen MS, Browne A, Hoffman GE, Stathopoulos S, Brennand K, Buxbaum JD, Drapeau E (2020) Transcriptional signatures of participant-derived neural progenitor cells and neurons implicate altered Wnt signaling in Phelan-McDermid syndrome and autism. Molecular Autism 11:53 32560742 10.1186/s13229-020-00355-0 PMC7304190

[31] Unno K, Chalmers ZR, Pamarthy S, Vatapalli R, Rodriguez Y, Lysy B, Mok H, Sagar V, Han H, Yoo YA, Ku SY, Beltran H, Zhao Y, Abdulkadir SA (2021) Activated ALK cooperates with N-Myc via Wnt/beta-Catenin signaling to induce neuroendocrine prostate cancer. Cancer Res 81:2157–2170 33637566 10.1158/0008-5472.CAN-20-3351 PMC8137566

[32] Rao S, Lungu C, Crespo R, Steijaert TH, Gorska A, Palstra RJ, Prins HAB, van Ijcken W, Mueller YM, van Kampen JJA, Verbon A, Katsikis PD, Boucher CAB, Rokx C, Gruters RA, Mahmoudi T (2021) Selective cell death in HIV-1-infected cells by DDX3 inhibitors leads to depletion of the inducible reservoir. Nat Commun 12:2475 33931637 10.1038/s41467-021-22608-z PMC8087668

[33] Zhao Y, Li Y, Zhang R, Wang F, Wang T, Jiao Y (2020) The role of erastin in ferroptosis and its prospects in cancer therapy. Onco Targets Ther 13:5429–5441 32606760 10.2147/OTT.S254995 PMC7295539

[34] Miotto G, Rossetto M, Di Paolo ML, Orian L, Venerando R, Roveri A, Vuckovic AM, Bosello Travain V, Zaccarin M, Zennaro L, Maiorino M, Toppo S, Ursini F, Cozza G (2020) Insight into the mechanism of ferroptosis inhibition by ferrostatin-1. Redox Biol 28:101328 31574461 10.1016/j.redox.2019.101328 PMC6812032

[35] Jing H, Song T, Cao S, Sun Y, Wang J, Dong W, Zhang Y, Ding Z, Wang T, Xing Z, Bao W (2019) Nucleotide-binding oligomerization domain-like receptor X1 restricts porcine reproductive and respiratory syndrome virus-2 replication by interacting with viral Nsp9. Virus Res 268:18–26 31132368 10.1016/j.virusres.2019.05.011 PMC7114581

[36] Wang C, Zeng N, Liu S, Miao Q, Zhou L, Ge X, Han J, Guo X, Yang H (2017) Interaction of porcine reproductive and respiratory syndrome virus proteins with SUMO-conjugating enzyme reveals the SUMOylation of nucleocapsid protein. PLoS ONE 12:e0189191 29236778 10.1371/journal.pone.0189191 PMC5728522

[37] Dong J, Zhang N, Ge X, Zhou L, Guo X, Yang H (2014) The interaction of nonstructural protein 9 with retinoblastoma protein benefits the replication of genotype 2 porcine reproductive and respiratory syndrome virus in vitro. Virology 464–465:432–440 25146601 10.1016/j.virol.2014.07.036 PMC7112046

[38] Livak KJ, Schmittgen TD (2001) Analysis of relative gene expression data using real-time quantitative PCR and the 2(-Delta Delta C(T)) Method. Methods 25:402–408 11846609 10.1006/meth.2001.1262

[39] Bei C, Zhang C, Wu H, Feng H, Zhang YA, Tu J (2023) DDX3X is hijacked by snakehead vesiculovirus phosphoprotein to facilitate virus replication via stabilization of the phosphoprotein. J Virol 97:e0003523 36744958 10.1128/jvi.00035-23 PMC9972964

[40] Gringhuis SI, Hertoghs N, Kaptein TM, Zijlstra-Willems EM, Sarrami-Forooshani R, Sprokholt JK, van Teijlingen NH, Kootstra NA, Booiman T, Dort KA, Ribeiro CM, Drewniak A, Geijtenbeek TB (2017) HIV-1 blocks the signaling adaptor MAVS to evade antiviral host defense after sensing of abortive HIV-1 RNA by the host helicase DDX3. Nat Immunol 18:225–235 28024153 10.1038/ni.3647

[41] Upadya MH, Aweya JJ, Tan YJ (2014) Understanding the interaction of hepatitis C virus with host DEAD-box RNA helicases. World J Gastroenterol 20:2913–2926 24659882 10.3748/wjg.v20.i11.2913 PMC3961968

[42] Wang W, Jia M, Zhao C, Yu Z, Song H, Qin Y, Zhao W (2021) RNF39 mediates K48-linked ubiquitination of DDX3X and inhibits RLR-dependent antiviral immunity. Sci Adv 7:eabe5877 33674311 10.1126/sciadv.abe5877 PMC7935364

[43] Kumar R, Singh N, Abdin MZ, Patel AH, Medigeshi GR (2017) Dengue virus capsid interacts with DDX3X-A potential mechanism for suppression of antiviral functions in dengue infection. Front Cell Infect Microbiol 7:542 29387631 10.3389/fcimb.2017.00542 PMC5776122

[44] Manning BD, Cantley LC (2007) AKT/PKB signaling: navigating downstream. Cell 129:1261–1274 17604717 10.1016/j.cell.2007.06.009 PMC2756685

[45] Engelman JA, Luo J, Cantley LC (2006) The evolution of phosphatidylinositol 3-kinases as regulators of growth and metabolism. Nat Rev Genet 7:606–619 16847462 10.1038/nrg1879

[46] Clevers H (2006) Wnt/beta-catenin signaling in development and disease. Cell 127:469–480 17081971 10.1016/j.cell.2006.10.018

[47] Dixon SJ, Lemberg KM, Lamprecht MR, Skouta R, Zaitsev EM, Gleason CE, Patel DN, Bauer AJ, Cantley AM, Yang WS, Morrison BR, Stockwell BR (2012) Ferroptosis: an iron-dependent form of nonapoptotic cell death. Cell 149:1060–1072 22632970 10.1016/j.cell.2012.03.042 PMC3367386

[48] Zhang H, Forman HJ (2012) Glutathione synthesis and its role in redox signaling. Semin Cell Dev Biol 23:722–728 22504020 10.1016/j.semcdb.2012.03.017 PMC3422610

[49] Stockwell BR, Friedmann AJ, Bayir H, Bush AI, Conrad M, Dixon SJ, Fulda S, Gascon S, Hatzios SK, Kagan VE, Noel K, Jiang X, Linkermann A, Murphy ME, Overholtzer M, Oyagi A, Pagnussat GC, Park J, Ran Q, Rosenfeld CS, Salnikow K, Tang D, Torti FM, Torti SV, Toyokuni S, Woerpel KA, Zhang DD (2017) Ferroptosis: a regulated cell death nexus linking metabolism, redox biology, and disease. Cell 171:273–285 28985560 10.1016/j.cell.2017.09.021 PMC5685180

[50] Jiang X, Stockwell BR, Conrad M (2021) Ferroptosis: mechanisms, biology and role in disease. Nat Rev Mol Cell Biol 22:266–282 33495651 10.1038/s41580-020-00324-8 PMC8142022

[51] Wang W, Green M, Choi JE, Gijon M, Kennedy PD, Johnson JK, Liao P, Lang X, Kryczek I, Sell A, Xia H, Zhou J, Li G, Li J, Li W, Wei S, Vatan L, Zhang H, Szeliga W, Gu W, Liu R, Lawrence TS, Lamb C, Tanno Y, Cieslik M, Stone E, Georgiou G, Chan TA, Chinnaiyan A, Zou W (2019) CD8(+) T cells regulate tumour ferroptosis during cancer immunotherapy. Nature 569:270–274 31043744 10.1038/s41586-019-1170-y PMC6533917

[52] Zhang Y, Swanda RV, Nie L, Liu X, Wang C, Lee H, Lei G, Mao C, Koppula P, Cheng W, Zhang J, Xiao Z, Zhuang L, Fang B, Chen J, Qian SB, Gan B (2021) mTORC1 couplescyst(e)ine availability with GPX4 protein synthesis and ferroptosis regulation. Nat Commun 12:1589 33707434 10.1038/s41467-021-21841-w PMC7952727

[53] Yang L, Fan Y, Zhang Q (2023) Targeting ferroptosis in renal cell carcinoma: potential mechanisms and novel therapeutics. Heliyon 9:e18504 37554789 10.1016/j.heliyon.2023.e18504 PMC10404959

[54] Kan X, Yin Y, Song C, Tan L, Qiu X, Liao Y, Liu W, Meng S, Sun Y, Ding C (2021) Newcastle-disease-virus-induced ferroptosis through nutrient deprivation and ferritinophagy in tumor cells. iScience 24:102837 34368653 10.1016/j.isci.2021.102837 PMC8326413

