# Supplementary figures and images for: PRRSV hijacks DDX3X protein and induces ferroptosis to facilitate viral replication
Source: Vet Res. 2024 Aug 18;55:103. doi: 10.1186/s13567-024-01358-y (PMC11331664; doi:10.1186/s13567-024-01358-y)

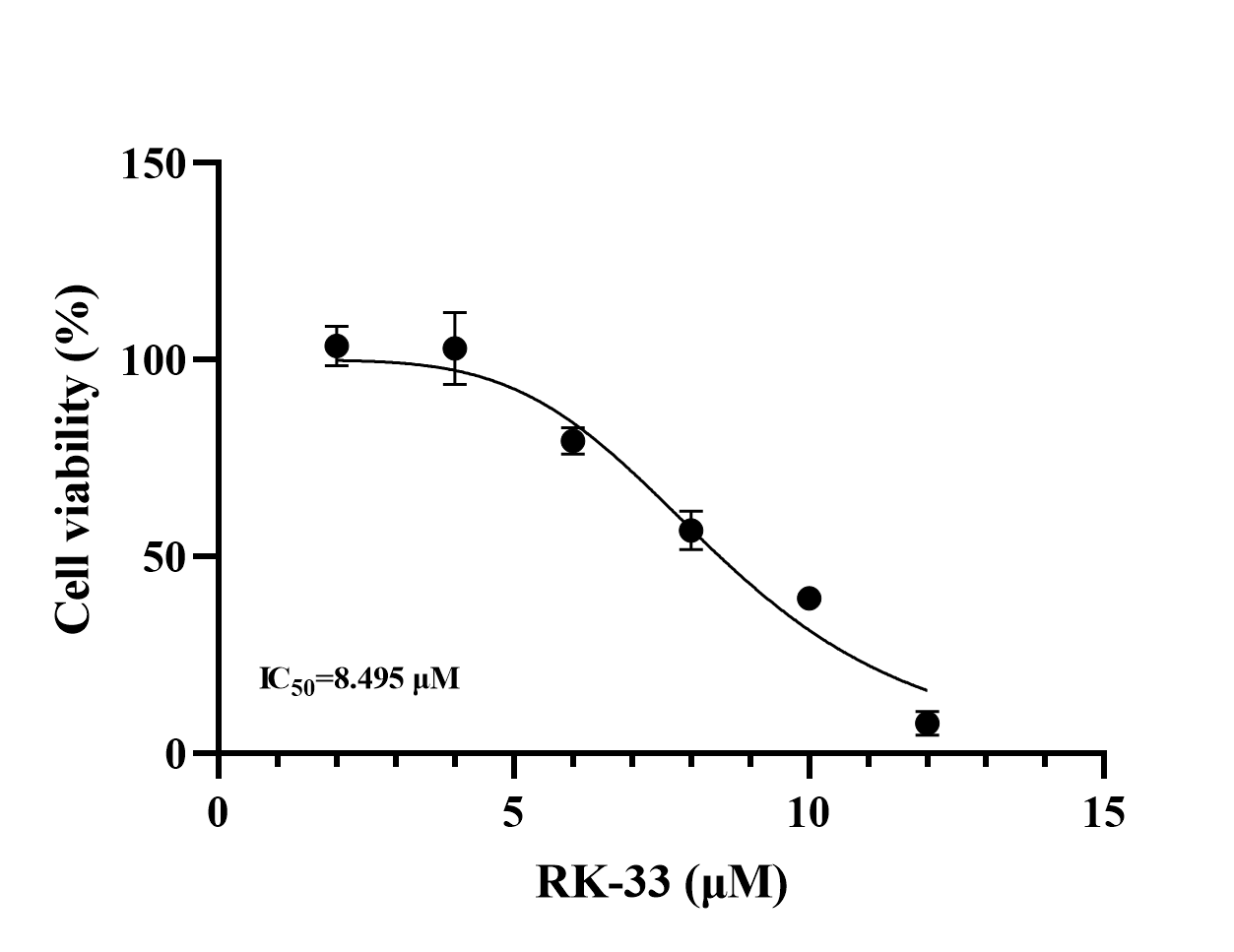

Supplement: Supplementary file 1 — Additional file 1. IC50. Marc-145 cells were treated with various concentrations of RK-33, and cell viability was determined using the CCK-8 assay. [file 13567_2024_1358_MOESM1_ESM.tif]

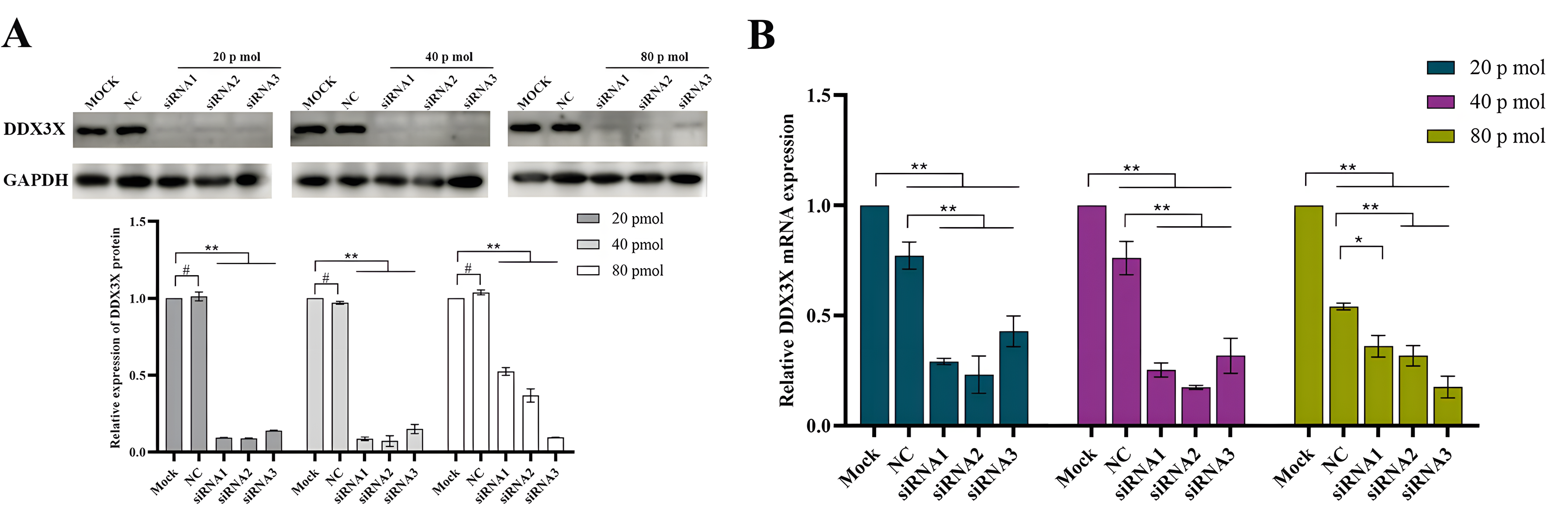

Supplement: Supplementary file 2 — Additional file 2. Evaluation of the effect of siRNA interference. Marc-145 cells, transfected with siRNA-DDX3X or siRNA-negative control (NC), were harvested and analysed by western blotting (A) and RT-qPCR (B). [file 13567_2024_1358_MOESM2_ESM.png]

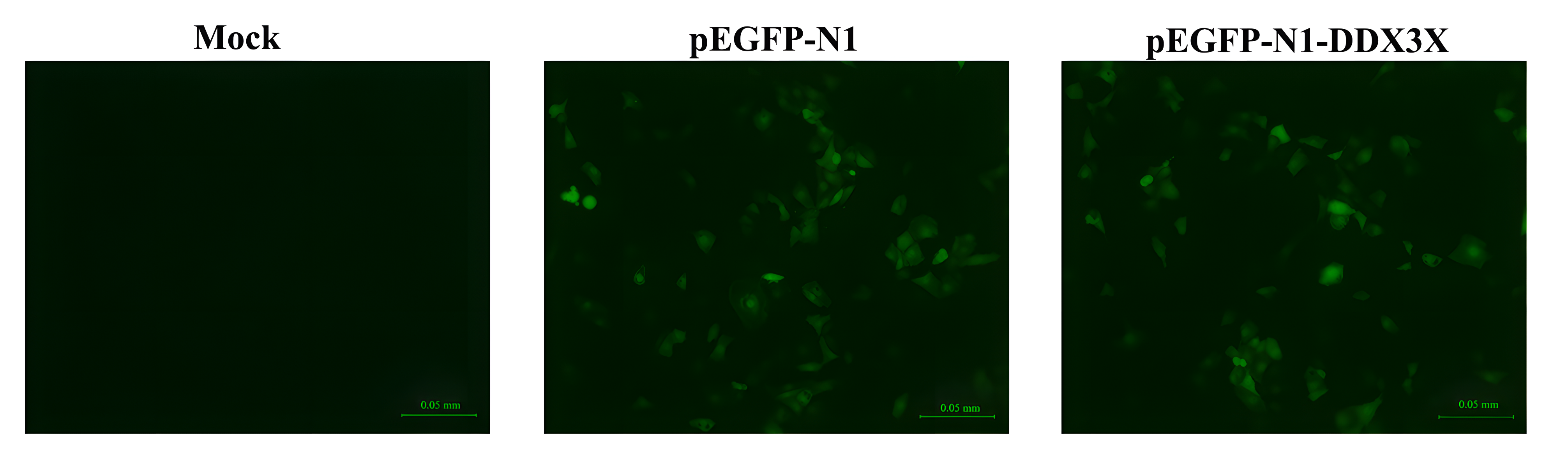

Supplement: Supplementary file 3 — Additional file 3. Validation of pEGFP-N1-DDX3X overexpression. Green fluorescence indicates successful transfection of DDX3X overexpression plasmid and control plasmid. [file 13567_2024_1358_MOESM3_ESM.png]
